# Supplementary material for: Heterogeneity analysis provides evidence for a genetically homogeneous subtype of bipolar-disorder
Source: ArXiv. 2024 Oct 27:arXiv:2405.00159v2. Originally published 2024 Apr 30. Preprint. [Version 2] (PMC11092873)
Supplement: Supplement 1 [file NIHPP2405.00159v2-supplement-1.pdf]

Supplementary Information (S1 Text)

This part contains a detailed description of our methods, including an outline of the steps involved and the considerations we made along the way. Also contains the supporting figures referenced in the main text.

1 Outline of analysis pipeline

In this section we briefly outline the major steps of our analysis pipeline. This includes (1) our initial biclustering analysis of the training arm-1, (2) our subsequent replication study on replication arms 2-4, and (3) the following PRS analysis on replication arms 2-4.

0. As a preliminary step, we requested individual level BD data from the PGC[1]. The data-set we requested includes the 27 cohorts listed in Fig 1. This dataset includes the raw genotyped and 1000-genomes European reference panel imputed data for more than $18K$ case-subjects and $29K$ control-subjects, as well as the associated genome-wide principal-components associated with each subject and used as a proxy for genetic ancestry. This data-set also includes metadata such as subtype information, which was not used to guide our analysis below.
1. In our primary analysis, we focused on the individual level raw genotyped data (i.e., we did not consider imputed data for the primary analysis).

[1]Currently, no permissions are required beyond a PGC BD group approval. We initially requested access in November, 2013. See `https://pgcdataaccess.formstack.com/forms/pgc_data_access_bip`

(a) First, we compared the available genotyped SNP lists from each cohort to divide the data into training and replication arms (see Fig 1 in the main text).

(b) We selected BDRN for our training arm-1. This was the largest single study available to us, containing 2524/4106 cases/controls, and allowed for a large replication arm-2 with 5781/8289 cases/controls sharing $\sim 85\%$ of the genotyped SNPs.

(c) We used an F-test to determine which of the genome-wide principal-components to include as proxies for ancestry in our primary analysis of arm-1.

(d) We then ran our biclustering algorithm on the raw genotyped data for the 2524 BD cases and 4106 controls in arm-1. We discuss this step in more detail within the next section. As a brief summary:

i. We included only the raw genotyped data with minor-allele-frequency (maf) $> 25\%$, and we corrected for case-control status as well as the genome-wide principal-components identified by the F-test above.

ii. We ran this algorithm with an 'elimination-fraction' of $\gamma = 0.5^8 \sim 0.004$, which is sufficient to ensure convergence (see supplementary Fig 32 in supplementary section 7.3 of [65]).

iii. We used a permutation test to determine the overall significance-level of the heterogeneity we observed. We found an overall significance level of $p \lesssim 1/64$, with the most prominent individual iterations in the range $i \in [175, 350]$.

2. After running our biclustering-algorithm on arm-1, we then used the structure of the bicluster to perform a replication-study in arms 2, 3 and 4.

(a) For each replication-arm, we determined which SNPs from the bicluster at iteration i were also present in that replication-arm.

(b) We then generated the dominant principal-component for the bicluster from arm-1 at iteration i, restricted to the SNPs in the intersection between arm-1 and the replication-arm. This principal-component is referred to as $v(i)$ in the main text.

(c) We then generated a 'bicluster-score' for each case- and control-subject by projecting the arm-1 and replication-arm data onto this dominant principal-component $v(i)$. In the main text we refer to these bicluster-scores in arm-1 as $u_{j_D}(i)$ and $u_{j_X}(i)$, for the cases and controls respectively. For the replication-arm we use the notation $u'_{j'_D}(i)$ and $u'_{j'_X}(i)$.

(d) We compute the covariate-corrected **AUC** for arm-1, denoted as $A(i)$, by comparing the values of $u_{j_D}(i)$ to the values of $u_{j_X}(i)$. Similarly, we calculate the covariate-corrected **AUC** for the replication-arm, denoted as $A'(i)$, by comparing the values of $u'_{j'_D}$ to those of $u'_{j'_X}$. These **AUC**-values are corrected for the seven covariates used in [20] (i.e., $\{U_1, \ldots, U_6\}$ as well as U_{19}).

(e) To assign a p-value to each covariate-corrected **AUC**-value we once again use a permutation-test involving random permutations of the case-control labels.

(f) After calculating these p-values, we concluded that the arm-1 bicluster-score indeed replicated across arms 2-4.

3. Given the results of the replication-study above, we switched from analyzing the raw genotyped data to analyzing the imputed data available for the subjects (see [20])

(a) We start by calculating, for each p-value threshold $\tilde{p}$, the values $\mathbf{PRS}_{\text{wide}}(j', \tilde{p})$ and $\mathbf{PRS}_{\text{bicl}}(j', i, \tilde{p})$ for each subject j', and bicluster iteration i where we see strong evidence of replication (i.e., for $i \in [175, 350]$).

i. To account for linkage-disequilibrium (LD), we use Plink's 'clump' function on the available imputed data for arm-1. We perform this clumping step using the same parameters as in [20] (i.e., info-score threshold of 0.9, R2-threshold of 0.1, genomic window of 500Kb, and minor-allele-frequency threshold of 0.05). We use the HRC EUR panel as our LD reference [70].

ii. For each p-value threshold $\tilde{p}$, we calculate each PRS-value for each individual as the sum of the risk-allele counts multiplied by the natural-log of the risk-allele odds-ratio. In this calculation we restrict the SNP-weight-vector to include only those SNPs with individual GWAS p-values that are more significant than the threshold $\tilde{p}$ (when forming the PRS).

A. For the $\mathbf{PRS}_{\text{wide}}(j', \tilde{p})$ values we use weights drawn from the summary statistics in [20] for arm-1 ('BDRN'). These summary statistics were generated by correcting for the genome-wide principal-components mentioned above (i.e., $\{U_1, \ldots, U_6\}$ and U_{19}).

B. For the $\mathbf{PRS}_{\text{bicl}}(j', i, \tilde{p})$ values we generate bicluster- and iteration-specific weights by running a covariate-corrected GWAS. For this calculation we restrict the case-subjects to the subset $\mathcal{J}(i)$ delineated by iteration i of the bicluster. However, we do not restrict the controls, and for this calculation we use all the controls from arm-1. Once again, to remain consistent with [20], we correct for the same genome-wide principal-components $\{U_1, \ldots, U_6\}$ and U_{19}.

(b) Once we have calculated $\mathbf{PRS}_{\text{wide}}(j', \tilde{p})$ on arms 2-4, we can consider the values of $\mathbf{PRS}_{\text{wide}}(j', \tilde{p})$ for a particular arm, comparing the results between cases and controls. We quantify this comparison by calculating a covariate-corrected **AUC** (correcting again for $\{U_1, \ldots, U_6\}$ and U_{19}) within this arm, denoted by $\mathbf{AUC}_{\text{wide}}(\tilde{p})$ in the main text.

(c) In a similar fashion we compare the case- and control-values of $\mathbf{PRS}_{\text{bicl}}(j', i, \tilde{p})$ by calculating the covariate-corrected **AUC** $\mathbf{AUC}_{\text{bicl}}(i, \tilde{p})$.

(d) By examining the values of $\mathbf{AUC}_{\text{wide}}(\tilde{p})$ and $\mathbf{AUC}_{\text{bicl}}(i, \tilde{p})$, as well as their dependence on $\tilde{p}$, we conclude that the bicluster-informed PRS-scores do a better job of highlighting SNPs that are useful for linear prediction (see Fig 8 in the main text).

(e) We then calculate $\mathbf{AUC}_{\text{wide}|\text{BDI}}(\tilde{p})$ and $\mathbf{AUC}_{\text{bicl}|\text{BDI}}(i, \tilde{p})$, restricting ourselves to a comparison between the case-subjects diagnosed with BDI and the control-subjects. This analysis indicates that the signal we saw across the replication arms in Fig 8 is likely carried by the BDI subjects (see Fig 9 in the main text).

(f) We then calculate $\mathbf{AUC}_{\text{wide}|\text{BDII}}(\tilde{p})$ and $\mathbf{AUC}_{\text{bicl}|\text{BDII}}(i, \tilde{p})$, restricting ourselves to a comparison between the BDII case-subjects and the control-subjects. This analysis indicates that the signal carried by the BDI subjects is not as strongly carried by the BDII subjects (c.f. Figs 9 and 10 in the main text).

(g) Finally, for each value of $\tilde{p}$, we pool the values of $\mathbf{PRS}_{\text{wide}}(j', \tilde{p})$ across all replication-arms, and recalculate the associated values of $\mathbf{AUC}_{\text{wide}}(\tilde{p})$, $\mathbf{AUC}_{\text{wide}|\text{BDI}}(\tilde{p})$ and $\mathbf{AUC}_{\text{wide}|\text{BDII}}(\tilde{p})$. Similarly, for each value of $\tilde{p}$ and i, we pool the values of $\mathbf{PRS}_{\text{bicl}}(j', i, \tilde{p})$ across all replication-arms and recalculate the associated values of $\mathbf{AUC}_{\text{bicl}}(i, \tilde{p})$, $\mathbf{AUC}_{\text{bicl}|\text{BDI}}(i, \tilde{p})$ and $\mathbf{AUC}_{\text{bicl}|\text{BDII}}(i, \tilde{p})$. We convert these **AUC**-values to R^2-values on a liability-scale (see [100]), producing $\mathcal{R}^{\mathbf{2}}_{\text{wide}}(\tilde{p})$, $\mathcal{R}^{\mathbf{2}}_{\text{wide}|\text{BDI}}(\tilde{p})$ and $\mathcal{R}^{\mathbf{2}}_{\text{wide}|\text{BDII}}(\tilde{p})$, as well as $\mathcal{R}^{\mathbf{2}}_{\text{bicl}}(\tilde{p})$, $\mathcal{R}^{\mathbf{2}}_{\text{bicl}|\text{BDI}}(\tilde{p})$ and $\mathcal{R}^{\mathbf{2}}_{\text{bicl}|\text{BDII}}(\tilde{p})$. We show the results in Fig 11. This analysis corroborates our conclusions above: the BDI subjects seem to be driving the bicluster-informed PRS-associated signal we see across the replication-arms.

2 Details regarding the biclustering method

Selection of minor-allele-frequency (maf) threshold for primary analysis:

In our primary analysis we restricted ourselves to maf$\geq$ 25% for the discovery arm. The main reason we did this was because we did not want our initial training to be too dependent on allele-combinations that were too rare. More specifically, the cohorts within the replication arms were of varying sizes, and several were relatively small (i.e., containing only a few hundred cases and controls). With an maf of only 05%, we would expect allele-combinations (e.g., homozygous recessive) with a prevalence of only $(1/20)^2 = 1/400$, and these allele-combinations might not even show up in the smaller cohorts. Thus, to be conservative and focus on allele-combinations that we would reasonably expect to see in a smaller study later on, we limited ourselves to maf$\geq$ 25%. With this larger maf of 25%, the rarest allele-combinations would have a prevalence of $(1/4)^2 = 1/16$, meaning that we should still expect to see a few dozen or so such combinations in each of the smaller cohorts.

Secondarily, we wanted to conduct our primary analysis (i.e., discovery in arm-1) with an eye towards the PRS-analysis we followed up with after our replication-study. This PRS-analysis involves imputed data, and there is evidence that imputation accuracy diminishes with maf (see [101]). Thus, restricting ourselves to SNPs with a sufficiently high maf also helps ensure that the SNPS we use to determine our signal will be accurately imputed in other data-sets.

With this being said, it is certainly reasonable to ask what kind of bicluster we would have found if we lowered the maf-threshold to maf$\geq$ 05% in arm-1. This lower maf-threshold produces a bicluster with many more SNPs (i.e., including many with maf ranging from 05% to 25%) but a strongly overlapping set of subjects. Intriguingly, the overlap in subjects (relative to chance) peaks within the iteration-interval $[175 - 350]$ and maintains highly significant enrichment (p in the range 10^{-35} to 10^{-45} throughout). We view this as corroboration that our selected range of iterations is a roughly accurate delineation of the true signal (see Fig 33).

In terms of next steps, it is quite natural to combine these (and similar) results with our primary biclustering analysis. By collecting the results of several biclustering analyses (each with different parameters), we could redefine the 'core' of the bicluster to be those case-subjects that lie in the intersection of the majority of trials (e.g., the case-subjects that lie in the intersection of the bicluster found using maf$\geq$ 25% and the bicluster found using maf$\geq$ 05%). One might also consider further restricting this core to include only those case-subjects that persist over the sensitivity-tests described below. We fully intend to pursue this strategy in future work; we have refrained from this approach in the current paper because our goal here is to describe a robust recipe that could be applied from scratch (without, e.g., knowing ahead of time which biclusters would and would not replicate).

Correcting for ancestry:

Before we run the biclustering method on a data-set, we need to choose how to correct for ancestry. Following the research of [20], we use genome-wide principal-components as a proxy for genetic ancestry. In [20], the genome-wide principal-components $U_1, \ldots, U_6$ and U_{19} were found to be significantly associated with case-control status across the studies considered. In our case we have fewer subjects in arm-1, and so we determined which genome-wide principal-components were significant for this arm by running an F-test applied to nested logistic regression on the sequence U_1, U_2, U_3 and so forth. This F-test selected only

U_1 and U_2 as significant for arm-1. Thus, when conducting our initial biclustering analysis in arm-1 we only correct for U_1 and U_2. However, when conducting our replication-study in arms 2, 3 and 4 we stay consistent with [20] and correct for components $U_1, \ldots, U_6$ and U_{19}. We also correct for $U_1, \ldots, U_6$ and U_{19} when conducting our PRS analysis later on.

Iterative biclustering-algorithm (recapitulated from the main text):

As mentioned in the main text, we use the half-loop method of [65]. We start by re-coding each SNP via its three allele-combinations: heterozygous and homozygous dominant and recessive. After recoding we'll assume the data-set contains M_D case-subjects and M_X control-subjects, each measured across N allele-combinations. We denote the array of case-subjects by D, with $D(j_D, k)$ referring to allele-combination-k in case-subject-j_D. Similarly, we denote the array of control-subjects by X, with $X(j_X, k)$ referring to allele-combination-k in control-subject-j_X. We use the generic subject-index j to refer to both the j_D and the j_X.

In its most basic form, the half-loop algorithm then proceeds as follows:

Step-0 First we load/initialize the data-arrays D and X.

Step-1 For each case j_D and allele-combination k, we measure the fraction of other cases in D which share that allele-combination, denoted by $[D \leftarrow D](j_D, k)$. Similarly, we measure the fraction of controls in X which share that allele-combination, denoted by $[D \leftarrow X](j_D, k)$. The difference between these two values, denoted by $Q(j_D, k) = [D \leftarrow D](j_D, k) - [D \leftarrow X](j_D, k)$ is a measure of the differential-expression of allele-combination k when contrasting that particular case-subject (at case-index j_D) with the control-population.

Step-2 After calculating $Q(j_D, k)$, we form the 'row-scores' $Q^{\text{row}}(j_D) = \sum_k Q(j_D, k)$. This row-score is a measure of the total differential-expression between case-index j_D and the control-population, accumulated across all allele-combinations. Similarly, we calculate the 'column-scores' $Q^{\text{col}}(k) = \sum_{j_D} Q(j_D, k)$. This column-score is a measure of the total differential-expression associated with allele-combination k, as measured between the case-population and the control-population. Finally, we calculate the overall 'trace' $\bar{Q} = \sum_{j_D, k} Q(j_D, k)$, which is a measure of the overall differential-expression exhibited between the case-patients in D and the controls in X, accumulated across all the allele-combinations in the data-array.

Step-3 At this point we remove a small fraction of those case-subjects and allele-combinations from D with the lowest row- and column-scores. For this analysis, the fraction we choose is $\gamma = 0.5^8 \sim 0.004$.

Step-4 We return to Step-1, iterating until there are no more case-subjects within D.

The biclustering-algorithm above proceeds iteratively; at each iteration i we remove a small fraction γ of the remaining case-subjects and allele-combinations. As described in supplementary section 7.3 in [65], the overall speed and accuracy of the biclustering-algorithm depends on γ. For large values of γ the algorithm is fast, but not particularly accurate. For smaller values of γ the algorithm becomes somewhat slower, but more accurate. In practice, the algorithm's detection-thresholds converge for values of $\gamma \lesssim 0.5^5 \sim 0.03$ (see Fig 32 in supplementary section 7.3 in [65]). Thus, in this analysis we make a conservative choice of $\gamma = 0.5^8 \sim 0.004$, which is roughly an order of magnitude smaller than the value required for convergence.

After each iteration i, we are left with a subset $\mathcal{J}(i)$ comprising $M(i)$ case-subjects and a subset $\mathcal{K}(i)$ comprising $N(i)$ allele-combinations. Together, these row- and column-subsets form an $M(i) \times N(i)$ sub-array $D(i)$ of the original D. If the case-array D were to contain a bicluster with a sufficiently strong signal, then the rows and columns of that bicluster would be retained until the end, with the other rows and columns eliminated earlier (see supplementary sections 2 and 15.2 of [65] for statistical guarantees).

This half-loop method has detection-thresholds similar to spectral-clustering and message-passing [68, 69], but has several additional useful features. First, the half-loop method allows us to search for disease-specific heterogeneity by directly correcting for control-subjects. This case-control correction also motivates the null-hypothesis H0 described below; the permutation-test allows us to avoid spurious structures that are unrelated to the disease-label. Second, the half-loop scores in Step-1 allow us to (implicitly) correct for linkage-disequilibrium (LD). More specifically, subsets of SNPs which are in equally strong LD in both the case- and control-populations will be excluded as the algorithm proceeds, unless some of those SNPs are involved in a pattern of differential-expression specific to the remaining case-subjects, in which case they will be retained (as desired). Third, the method also allows us to correct for continuous covariates. This covariate-correction is described in detail in supplementary section 10 of [65], but essentially amounts to a reweighting of the $Q(j, k)$ in Step-1 to reduce the overall level of differential-expression contributed by structures which are not evenly distributed in covariate-space. Finally, the method itself is rather straightforward and does not require the fine-tuning of parameters; the accuracy of the algorithm converges as $\gamma \to 0$, and as long as γ is sufficiently small the detection thresholds for the algorithm will not depend on γ.

Identifying biclusters:

As described above, our algorithm for biclustering proceeds as follows:

First, we run the biclustering algorithm on the original data-set. As the algorithm proceeds through its iterations, it produces a nested sequence of subsets $\mathcal{J}(i)$ and $\mathcal{K}(i)$ (with i referring to the iteration-index). Along the way we record the trace $\bar{Q}(i)$, which is a covariate-corrected measure of the overall level of differential expression between the remaining case-subjects (i.e., $\mathcal{J}(i)$) and the controls, restricted to the allele-combinations within $\mathcal{K}(i)$. This trace is shown as the red-curve in Fig 2, and does not by itself pick out any particular bicluster, nor any particular iteration.

We determine if there is sufficient statistical evidence for a bicluster by performing a permutation-test: We randomly permute the case-control labels of that data-set to generate random trials from a null-hypothesis. This null hypothesis (referred to as $H0$ in the main text) corresponds to the hypothesis that any heterogeneity in the data-set is not linked to case-control status. As a technical detail, we preserve the covariate-structure determined by the genome-wide principal-components within this permuted data by permuting case-control labels while respecting proximity in the covariate-space (see supplementary section 10 in [65]). We then rerun the biclustering algorithm on all these label-shuffled data-sets, producing a trace for each label-shuffled trial drawn from $H0$ (see black curves in Fig 2 in the main text). We then compare the original trace (red) to the label-shuffled traces (black), and measure the overall level of significance (see supplementary section 14.2 in [65]).

For a typical 'noisy' data-set, this first biclustering run would not return a significant overall p-value, and we would stop, concluding that there is not sufficient evidence for heterogeneity in the form of a bicluster. However, if (and only if) the original trace is significant, we can conclude that there is sufficient evidence for heterogeneity in the form of a bicluster. This is the case for arm-1 (with the overall p-value $\lesssim 1/64$).

The actual 'boundary' of this bicluster is then determined by comparing the original trace with the label-shuffled traces (e.g., looking for iterations where the difference is significant). In some cases the red-trace will have a distinguished maximum, clearly indicating an iteration i which can be used to cleanly delineate the bicluster (i.e., comprising $\mathcal{J}(i)$ and $\mathcal{K}(i)$). However, in many real situations (such as this analysis), the scenario is less straightforward. While the overall difference between the red- and black-traces was significant, there was no clear maximum towards the middle of the red-trace. Rather, there was a 'plateau' extending across a range of iterations $i \in [175, 350]$. Thus, in order to be conservative, we thought of the first bicluster as a 'fuzzy' bicluster defined by a subset of case-subjects somewhere in-between $\mathcal{J}(350)$ and $\mathcal{J}(175)$ and a subset of allele-combinations somewhere in-between $\mathcal{K}(350)$ and $\mathcal{K}(175)$. Recall that these are nested in one another, with $\mathcal{J}(350) \subset \mathcal{J}(175)$ and $\mathcal{K}(350) \subset \mathcal{K}(175)$.

Searching for multiple biclusters:

Only once we believe that there is sufficient statistical evidence for a first bicluster do we search for a second. To perform this second search, we scramble the entries in the bicluster identified from the original trace and then repeat our biclustering-algorithm on this scrambled data-set, attempting to search for a second bicluster. In this manner, the search for the second bicluster requires us to delineate the first bicluster (otherwise the search for a second bicluster would return exactly the same results as the first search). In our case, just to be conservative, we scrambled the entries in the bicluster delineated by $\mathcal{J}(163)$ and $\mathcal{K}(163)$, which includes all the case-subjects and allele-combinations that could have fallen into the fuzzy range of our first-bicluster.

If this second bicluster were also significant, we would have searched for a third, and so forth. As illustrated in Figs 2 and 25, when we used this strategy to search arm-1 for case-biclusters we only found a single significant bicluster: the second case-bicluster in arm-1 was only marginally significant (i.e., an overall p-value of ~ 0.05), and so we stopped our search there. In principle we could have searched for a third bicluster, but because this second bicluster failed to replicate in arms 2, 3 and 4 (see Figs 26-28), we did not continue.

When searching for the control-biclusters in arm-1 our first trace was not significant (Fig 29), and so we stopped our search (i.e., we never searched for a second bicluster within the control-subjects in arm-1). However, when searching for control biclusters in arms 2, 3 and 4, we actually found several significant control-biclusters. Many of these control-biclusters then replicated in the other arms (see, e.g., Figs 30-32 for the first control-bicluster found in each of these other arms). However, we elected to defer study of these control-biclusters to a later paper, as we already had enough to report on from the case-bicluster in arm-1.

Incidentally, we also searched arms 2, 3 and 4 for case-biclusters (i.e., treating each as a discovery-arm). In each case there was some evidence for significant case-biclusters, with particularly strong evidence in arms 3 and 4. The traces for these biclusters are illustrated in Figs 43-45. For this paper we elected not to pursue these biclusters, mainly because of the evident heterogeneity in the control-populations for these arms. To elaborate, the reason we were wary is that our biclustering algorithm is designed to work most cleanly in an environment where the control-subjects are a well-sampled representation of the true control-population (as this is where we can guarantee the statistical bounds described in sections 18-22 of the supplementary information in [65]). Because the controls in arms 2, 3 and 4 are not homogeneous, it is possible that there is batch-specific structure to the control-heterogeneity that is not directly related to disease-label. For example, one of the control cohorts (bmpo) was recruited from a cancer study. Thus, even though we corrected our search for the case-population and the resulting control-biclusters are significantly related to control-case label, it might be the case that this association is driven by recruitment artifacts or covariates that we don't have access to. This potential for an unknown (but structured) source of heterogeneity for the controls in arms 2, 3 and 4 made us hesitant to pursue the case-biclusters found in these arms in this paper. However, we intend to investigate these structures in future work.

Sensitivity:

Our biclustering method (taken from [65]) is more-or-less automatic, and once the experimental design is determined (i.e., the arrangement of cases, controls and covariates) and a sufficiently small elimination-fraction γ is chosen, there aren't any additional parameters that can be tweaked. Nevertheless, we can still perform a kind of sensitivity analysis by deliberately forcing the algorithm to produce a different output by changing which subjects and SNPs are excluded at each iteration. One strategy for such a sensitivity analysis is to force the loop-counting method to exclude subjects and SNPs one at a time – recalculating the loop-scores after each individual exclusion. This dramatically slows down the overall algorithm, but can force the subjects and SNPs to be excluded in a different order (particularly during the first few iterations at the beginning).

When we perform this sensitivity test we find that, indeed, the subjects and SNPs are excluded in a different order. However, largely the same subset of subjects were retained during the iterations corresponding to the interval highlighted in Figs 3-5. More specifically, the range we highlight in Figs 3-5 corresponds to retaining 47% – 21% of the original case-subjects. When we perform our sensitivity test and look at the iterations for which the number of case-subjects fall into this same range, we find an overlap of $\sim 75\%$ with our original subset, a very significant enrichment ($p \leq 10^{-80}$, see Fig 34).

As described above in the discussion regarding minor-allele-frequency, we believe it's an excellent idea to use these kinds of sensitivity-tests to whittle down the bicluster its 'core'. For example, we could retain only those case-subjects that persist across a variety of sensitivity-tests. As mentioned above, we refrained from doing this for the present paper because we wanted our analysis pipeline to be simple to understand, and we didn't want to add extra hyperparameters (such as how many and what kinds of sensitivity-tests to run, how to intersect the various biclusters found across these sensitivity tests, and so forth).

Overfitting:

Overfitting is a very real problem, particularly when attempting to characterize heterogeneity. In our case we have tried our best to avoid overfitting by adopting the following safeguards.

First, we use a permutation-test to characterize the significance of the original biclustering-algorithm (within the discovery-process in arm-1), as described above and illustrated in Fig 2. Briefly, this permutation-test involves running the same biclustering-algorithm (with the same parameters, principal-components, etc.) but on a randomly permuted copy of arm-1 for which the case-control labels were shuffled. Thus, much of the structure and correlations in this label-shuffled data matches that of the original data; the main difference being that the disease-label no longer correlates with the disease. By comparing the trace $\bar{Q}(i)$ for each iteration of the original biclustering run (red curve in Fig 2) against the distribution of traces obtained from the shuffled-data (grey curves in Fig 2), we can determine an overall p-value for the entire biclustering process. If this overall p-value were not significant, we would have stopped and not pursued any further analysis. However, in the case of our first biclustering-run, this overall p-value was $\lesssim 1/64$, giving us confidence that there was indeed a significant signal linking the bicluster to the disease-labels in arm-1.

Now, while we believe that our first bicluster from arm-1 is a significant signal, we can't be sure that this bicluster is disease-related, as there could be some problem with the labels themselves (e.g., a batch-effect we were unaware of). Thus, as a second safeguard against overfitting, we check for replication in three independent data-sets. That is to say, we used the bicluster to define bicluster-scores, and then checked to see if the bicluster-scores retained information related to the disease-label in the replication-arms (i.e., Figs 3-5).

This second safeguard helped us reassure ourselves that the bicluster we found was indeed linked to both the disease-label as well as the SNPs selected (and unlikely to be entirely driven by an unaccounted-for covariate). However, the bicluster could still be driven by some idiosyncrasy within the genotyping process, and might not generalize to other genotyping platforms. For example, the replication in Figs 4 and 5 is not as strong as that in Fig 3; we currently believe that this phenomenon is due to the fact that the number of genotyped SNPs shared between arm-1 and arm-2 is greater than the number of genotyped SNPs shared between arm-1 and arms-3 and 4. However, it is also possible that this phenomenon occurs because our bicluster is platform-specific, and does not extend to data genotyped on other platforms.

Thus, as a third safeguard, we performed the PRS-analysis illustrated in Figs 7 and 8. This analysis is not as strongly tethered to exactly which SNPs were genotyped. Indeed, since we are able to use imputed data to conduct the PRS-analysis, we conducted this analysis using all SNPs available with minor-allele-frequence $\geq 05\%$. Thus, the number of accessible SNPs for this analysis is comparable across arms 2, 3 and 4. Put another way, the PRS-analysis is not directly hampered by a reduction in overlap-fraction, and arms 3 and 4 seem to have the potential to replicate just as well as arm-2 (see, e.g., the $i = 225$ subplot within Fig 8). We believe that this PRS-analysis helps strengthen our claim that this bicluster is not an artefact of the genotyping platform for arm-1, and is actually selecting a subset of patients that are correlated in a disease-relevant manner.

Comparison to other methods:

As far as we are aware, our biclustering method is the only available open-source software we know of which can tackle large arrays of categorical data (i.e., hundreds of thousands to millions of genotyped SNPs) while correcting for cases and controls (i.e., disease status), and also correcting for continuous covariates (i.e. genome-wide principal-components). Thus, we were not able to apply another biclustering method to the genotyped bipolar data. However, we have certainly used other data-sets to compare our biclustering method to other methods in the literature.

Originally, we used synthetic data-sets to compare the performance of our biclustering algorithm to several other algorithms (see supplementary section 7.4 in [65]). As demonstrated in this paper, our method performs quite favorably, with higher accuracy than the classical methods tested.

More recently, we have applied our biclustering methods to snRNA-seq data, and we have compared the performance of our method against two popular methods in the literature (UMap and Louvain-clustering). These comparisons (involving both simulated and real data) are described in the appendix S1 of [102]. In summary, we find that both UMap and Louvain-clustering have a difficult time determining which clusters/biclusters are statistically significant, and which are spurious. By contrast, our approach uses the permutation-test described above to estimate the overall p-value of any potential bicluster. Moreover, as described above, we are quite conservative in our approach, searching for biclusters one at a time, and only proceeding if the previous searches return statistically significant results. As a result of these safeguards our biclustering method is much more accurate than UMap and Louvain-clustering at recovering biologically relevant structure.

Possible limitations:

One limitation of our methodology is that, so far, we have only used the raw genotyped data as input to detemine biclusters in our training arm-1. We did so to avoid concerns of spurious correlations that might manifest within imputed data [60]. However, this choice limited the range of SNPs available for our replication-study in Figs 3-5. Going forward, we intend to extend our approach to allow for imputated data within the training-arm. In the best case scenario this imputed data will be fully accurate and will not introduce any spurious correlations. More generally, we will need to characterize – and correct for – any spurious structure introduced by the imputation. Doing so will open the door for higher powered analyses. Additionally, if we are able to deduce genetic subgroup membership directly from high-quality imputed data, then we will have even more options available for replication and validation.

Another shortcoming of our method is that it is likely *too* conservative when it comes to identifying heterogeneity. As an example, when studying Alzheimer's disease we encountered scenarios where there was clearly heterogeneity present, but the metric we used to compare the original-trace to the label-shuffled traces was not sensitive enough to pick out the nature of this heterogeneity (see, e.g., the section entitled "Search for disease-specific biclusters" and Figure S4 in [103]). In this study we suspect that the heterogeneity in the data is more complicated than can be characterized by a simple bicluster. That is, the heterogeneity may involve structures that are more nuanced than a distinguished set of subjects and SNPs (see section 7.3 and Fig 35 in [65]), and we would need a more sophisticated strategy to identify it. We believe that something similar is happening with the second case-bicluster found in arm-1 (see Fig 25), and we intend to pursue this line of inquiry in future work.

3 Additional supporting information and supporting figures

Additional replication analyses in arm-2

Figs 13 and 14 illustrate the replication observed in arm-2 when the SNP-overlap between arm-1 and arm-2 is artificially decreased.

Influence of covariates on the bicluster

Figs 15 and 16 illustrate the association between the bicluster found in arm-1 and the ancestry-related covariates. Fig 17 illustrates the association with sex.

Interaction between bicluster-score and population-wide PRS

As described in the Methods section, we calculated the population-wide $\mathbf{PRS}_{\text{wide}}(j';\tilde{p})$ and the bicluster-informed $\mathbf{PRS}_{\text{bicl}}(j';i,\tilde{p})$ across a variety of iterations i and $\tilde{p}$-thresholds. In Fig 18 we illustrate the correspondence between the population-wide $\mathbf{PRS}_{\text{wide}}(j';\tilde{p})$ and bicluster-score $u'_{j'}(i)$ for arm-2 at $i = 175$. This trend persists for other iterations, as illustrated in Figs 19 and 20.

Nagelkerke R^2 for a series of linear-models

To complement the observations of Figs 18, 19 and 20, we investigate how the bicluster-informed PRSs and the bicluster-scores themselves contribute to risk-prediction. For a given i and $\tilde{p}$ we build a series of linear-models to predict case-control status. These linear-models use: (i) the ancestry-related covariates, (ii) the population-wide $\mathbf{PRS}_{\text{wide}}(j';\tilde{p})$, (iii) the bicluster-informed $\mathbf{PRS}_{\text{bicl}}(j';i,\tilde{p})$, and (iv) the bicluster-score $u'_{j'}(i)$.

The series of linear-models we consider involve successively more and more of these terms. Thus, the null linear-model attempts to predict case-control status using no terms at all (i.e., using only the average prevalence of cases and controls). The first linear-model attempts to predict case-control status using only term (i), that is, only the ancestry-related covariates. The second linear-model uses both term (i) and (ii), i.e., both the ancestry-related covariates and the population-wide PRS. The third linear-model uses terms (i), (ii) and (iii). And finally, the fourth linear-model uses terms (i)-(iv). For each linear-model

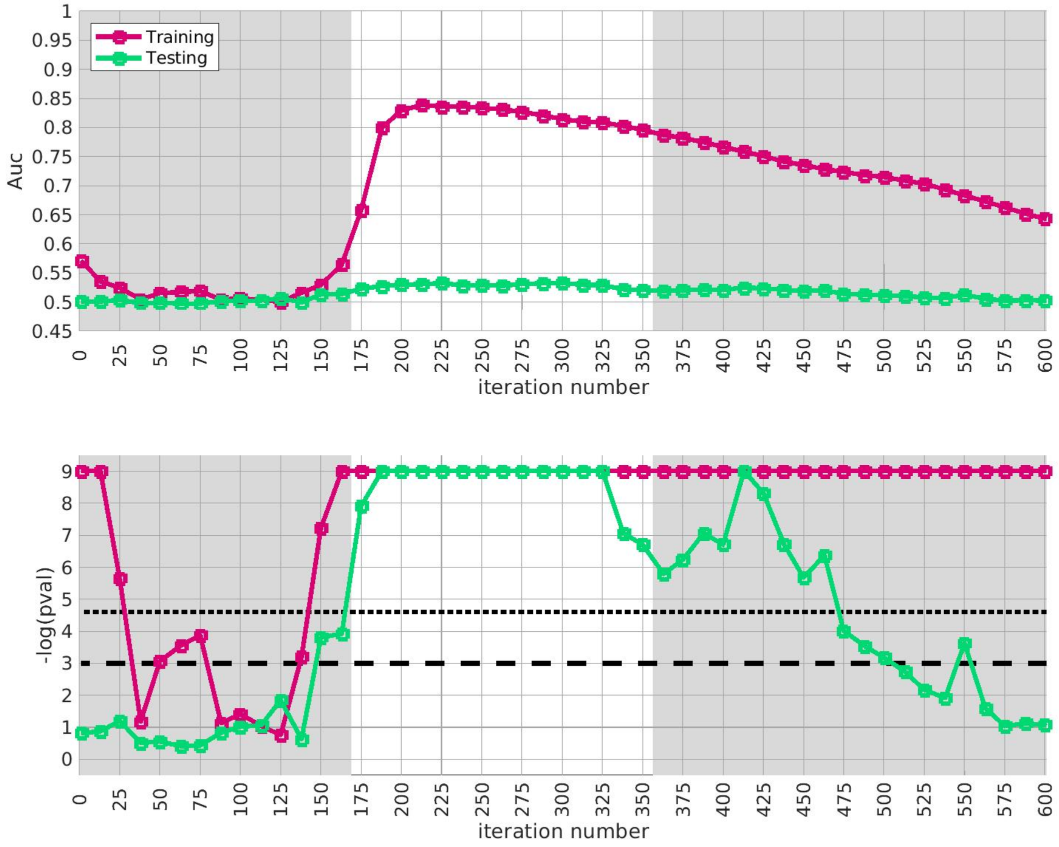

Figure 13: This figure is similar to Fig 3, except that we randomly eliminate SNPs from arm-2 until the SNP-overlap between arm-2 and the training-arm is equal to the SNP-overlap between arm-3 and the training-arm.

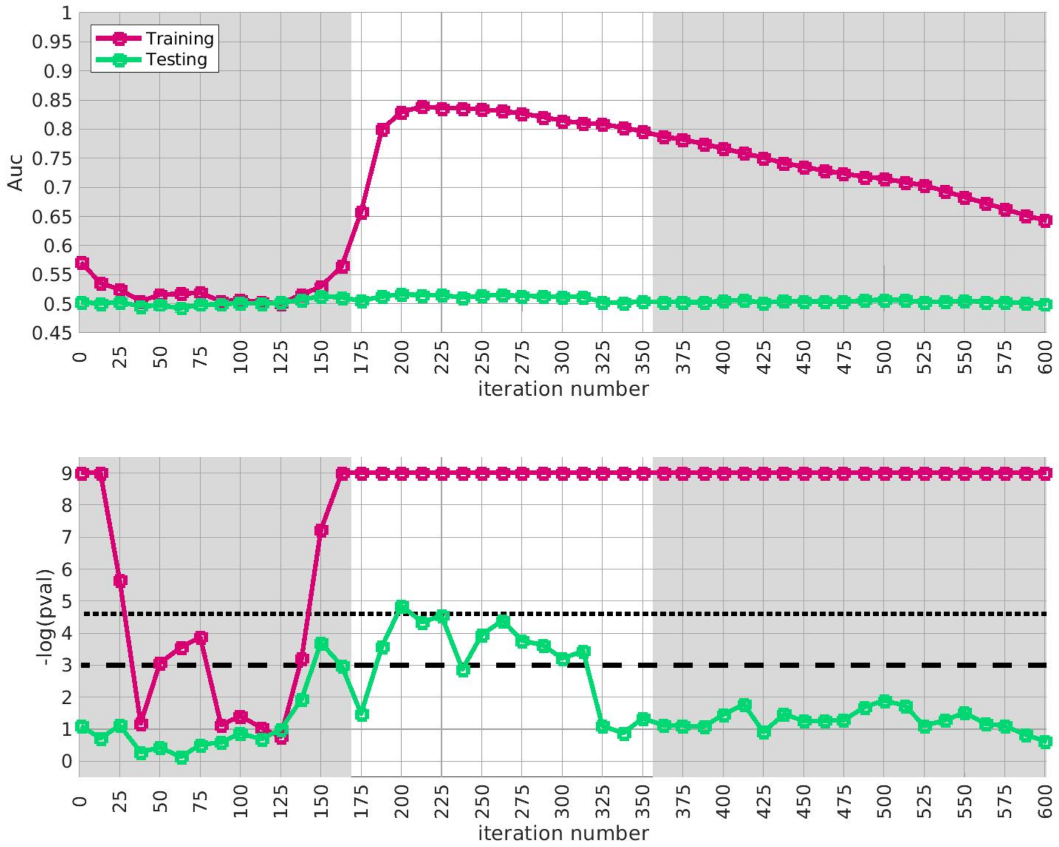

Figure 14: This figure is similar to Fig 3, except that we randomly eliminate SNPs from arm-2 until the SNP-overlap between arm-2 and the training-arm is equal to the SNP-overlap between arm-4 and the training-arm.

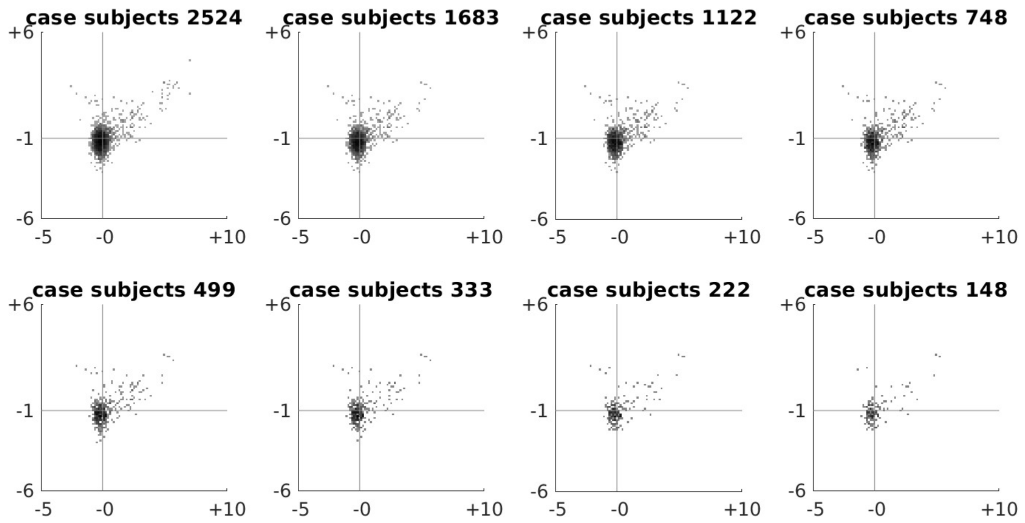

Figure 15: This figure illustrates the distribution of ancestry-associated principal-components U_1^1 and U_2^1 amongst the remaining case-subjects as the half-loop algorithm proceeds (specifically, at iterations $i \in \{96, 189, 276, 359, 442, 511, 585\}$). Each subplot displays a scatterplot of remaining case-subjects plotted with respect to U_1^1 and U_2^1. The number of remaining case-subjects is shown above each subplot. The horizontal and vertical lines indicate the median values for the original distribution. Note that the overall shape of the distribution does not change much as the algorithm proceeds.

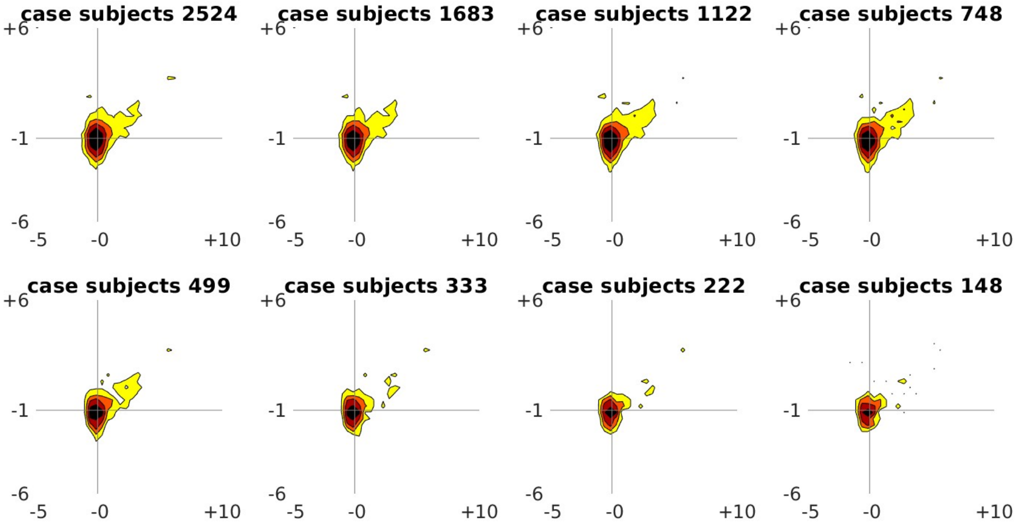

Figure 16: This figure is similar to Fig 15, except that a heat-map of the distribution is shown (rather than a scatterplot). The colors in the heat-map correspond to the logarithm of the density in the underlying distribution. Four different contours are shown, ranging from yellow to maroon, corresponding to the 20%, 40%, 60% and 80% percentiles of the log-density.

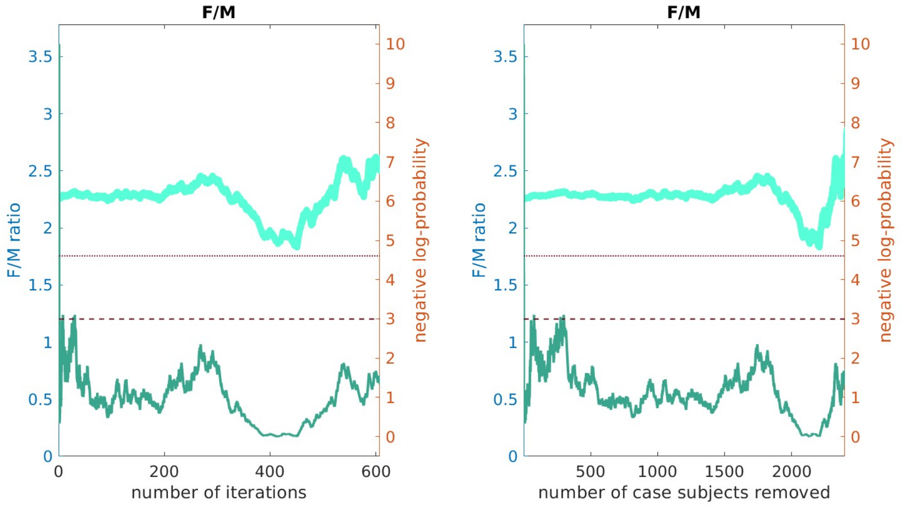

Figure 17: This figure plots the ratio of female to male subjects within $\mathcal{J}(i)$ (light-teal, left y-axis) as a function of the iteration i (left) and the number of remaining case-subjects (right). The dark-teal line corresponds to the negative-log-probability (right y-axis) of observing a ratio at least as large by chance. The dashed and dotted horizontal lines indicate 0.05 and 0.01 significance values, respectively. Note that the female population is not over-represented across the range of iterations including $i \in [175, 350]$, implying that the bicluster we observe is not significantly enriched for female subjects.

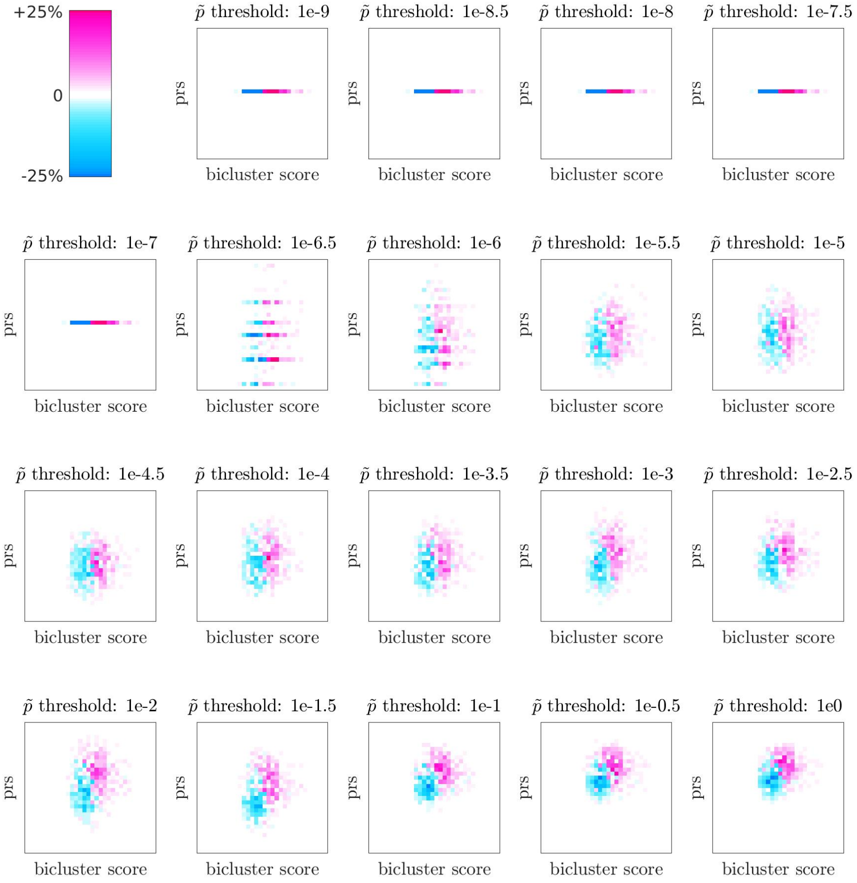

Figure 18: In this figure we illustrate the correspondence between the population-wide $\mathbf{PRS}(j';\tilde{p})$ and bicluster-score $u'_{j'}(i)$ for arm-2 at $i = 175$. Each subplot visualizes the distribution of subjects in arm-2 as a function of bicluster-score $u'_{j'}(i)$ (horizontal) and $\mathbf{PRS}_{\text{wide}}(j';\tilde{p})$ (vertical), with the SNP-p-value threshold $\tilde{p}$ varying across the subplots. In each subplot a heatmap is shown, representing the difference between the density of cases and controls. The color pink corresponds to areas with a higher case-density than control-density, while blue corresponds to areas with a higher control-density than case-density. The colorbar (upper-left) ranges across $\pm 25\%$ of the maximum density (taken across both the case- and control-distributions). Note that, while the bicluster-score is correlated with the population-wide PRS when $\tilde{p}$ is sufficiently high, the correlation is far from perfect. Note that, when $\tilde{p}$ is high (i.e., $\tilde{p} \sim 1$ and all the SNPs are used to generate the PRS), there is a marked correlation between case-control status and high-values of $\mathbf{PRS}_{\text{wide}}(j';\tilde{p})$ and bicluster-score $u'_{j'}(i)$. However, for lower values of $\tilde{p}$ this structure shifts, and $\mathbf{PRS}_{\text{wide}}(j';\tilde{p})$ is no longer a useful indicator of case-control status while the bicluster-score $u'_{j'}(i)$ is still a useful indicator. This trend persists for other iterations, as illustrated in Figs 19 and 20.

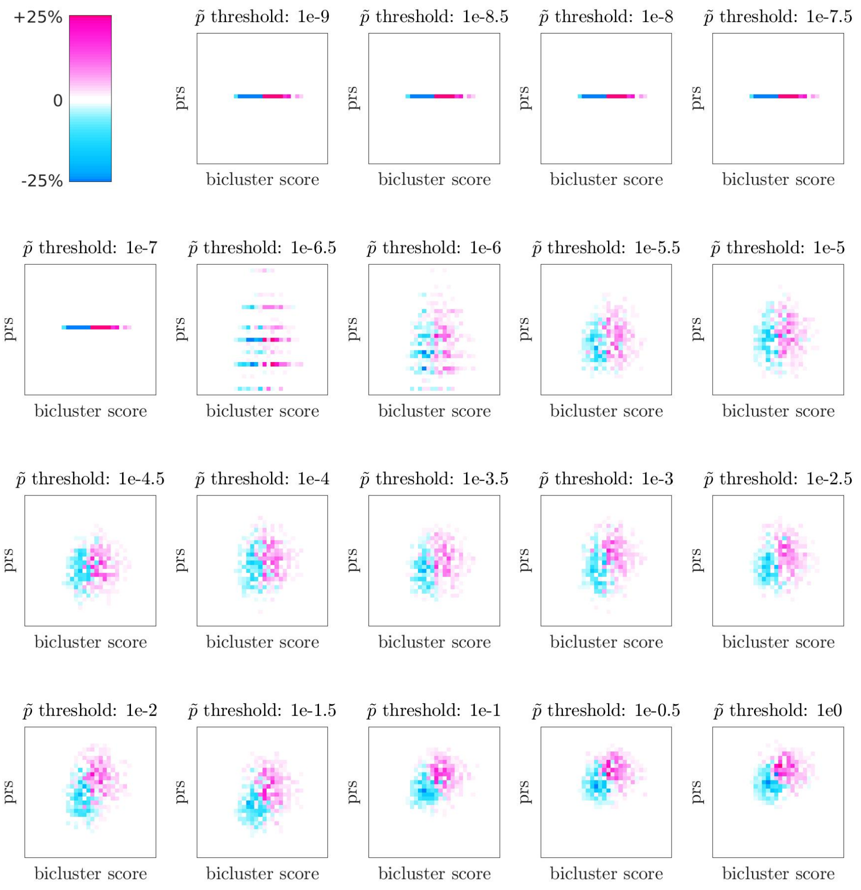

Figure 19: This figure is analogous to Fig 18, except for $i = 225$.

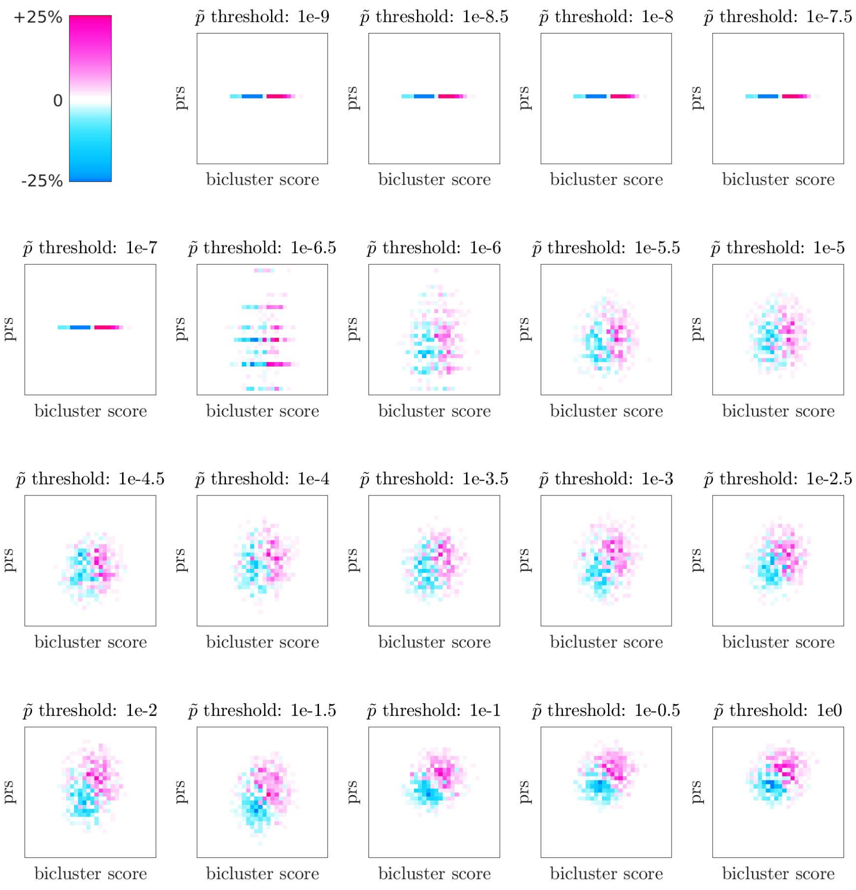

Figure 20: This figure is analogous to Fig 18, except for $i = 350$.

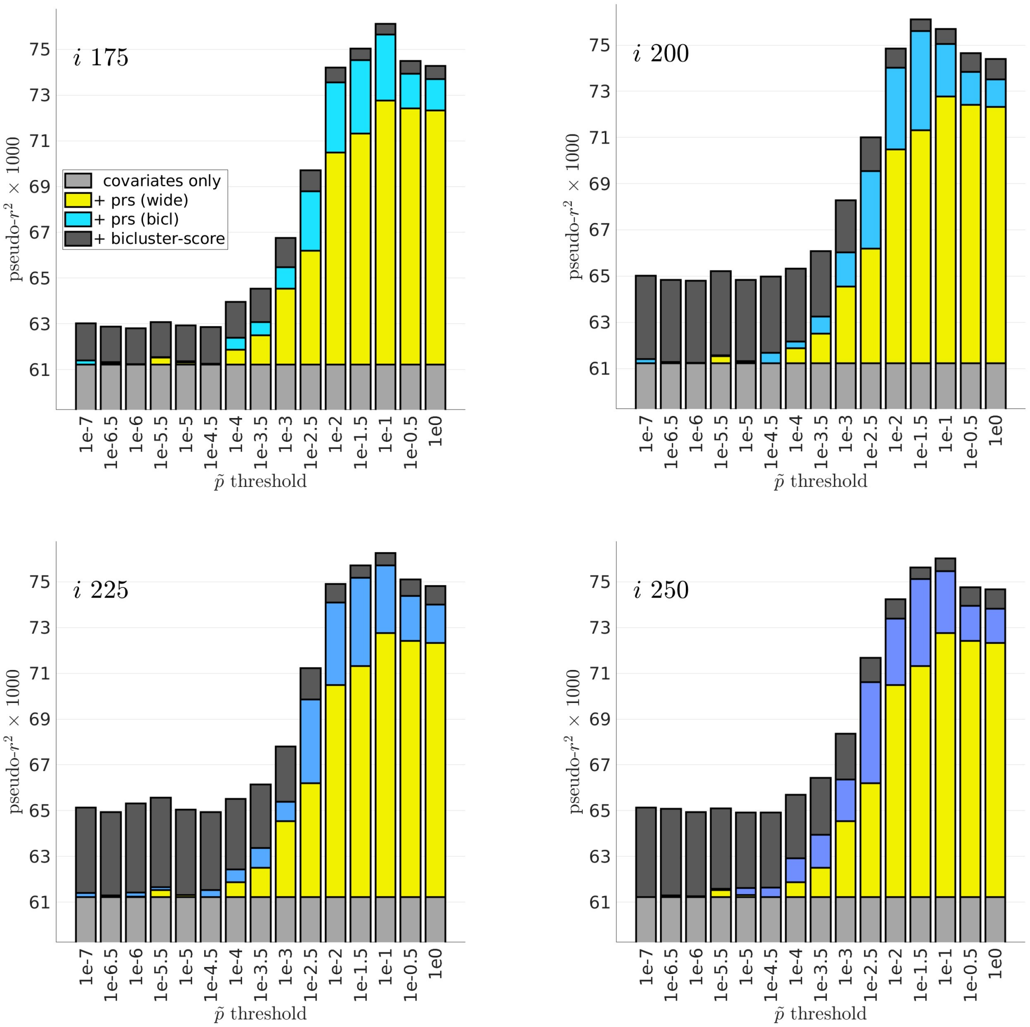

Figure 21: In this figure we illustrate the explanatory power of the bicluster-informed $\mathbf{PRS}_{\text{bicl}}(j'; i, \tilde{p})$ in combination with the bicluster-score $u'_{j'}(i)$ for arm-2, with the iteration number i varying across the subplots. Within each subplot the $\tilde{p}$-threshold is shown along the horizontal-axis. We measure the incremental Nagelkerke pseudo-r^2 value associated with the following terms: (i) the ancestry-related covariates, (ii) the population-wide $\mathbf{PRS}_{\text{wide}}(j'; \tilde{p})$, (iii) the bicluster-informed $\mathbf{PRS}_{\text{bicl}}(j'; i, \tilde{p})$, and (iv) the bicluster-score $u'_{j'}(i)$. For each i and $\tilde{p}$ we show a vertical bar divided into segments illustrating the contribution of each term (i)-(iv), with colors light-grey, yellow, blue-pink and dark-grey (respectively). The colors used for the third term (ranging from blue to pink) correspond to the colors used in Fig 7 below.

we measure the Nagelkerke pseudo-r^2 value between that model and the null model The difference in pseudo-r^2 values gives an estimate of the additional explanatory power provided by each term in succession.

Results for various i and $\tilde{p}$ are shown in Figs 21 and 22. Each vertical bar in this figure corresponds to a particular i and $\tilde{p}$. Each vertical bar is further divided into segments illustrating the incremental Nagelkerke pseudo-r^2 value associated with each term. Note that the bicluster-informed PRS, as well as the bicluster-score $u'_{j'}(i)$ add explanatory power to the underlying linear model. This phenomena is most pronounced when i is in the middle of the range $i \in [175, 350]$ and the threshold $\tilde{p}$ is small.

The observations in Figs 18-22 suggest that the bicluster might contain information useful for improving risk-prediction. Moroever, the peak in overall R^2 when $\tilde{p} \sim 1e-2$ suggests that the signal in our bicluster-score involves subsets of SNPs which do not individually achieve genome-wide significance.

Replication results for arm-3 and arm-4

Figs 23 and 24 illustrate the replication analyses on arms 3 and 4.

Significance of secondary bicluster in arm-1

Figs 25 26 27 and 28 illustrate the significance of the secondary bicluster found in arm-1.

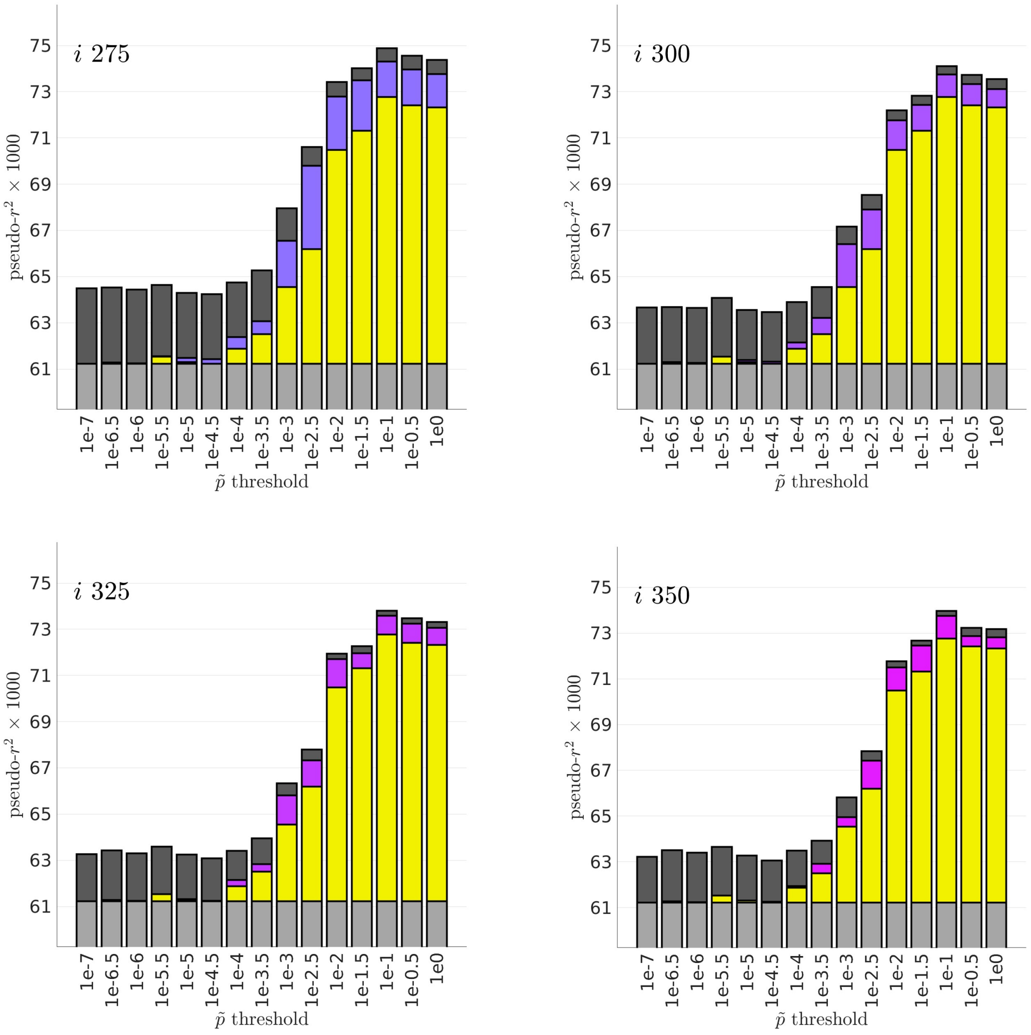

Figure 22: This is analogous to Fig 21, for a different set of iterations.

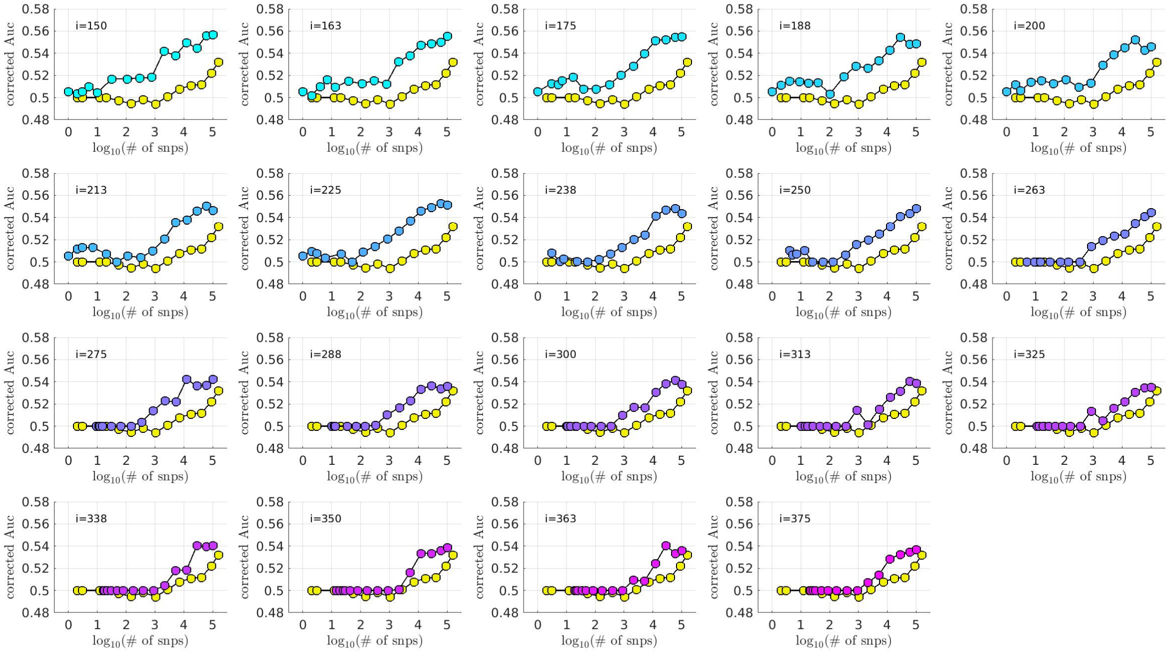

Figure 23: This figure is analogous to Fig 7, except that we test on arm-3 rather than arm-2.

Control-specific biclusters

Figs 29, 30, 31 and 32 illustrate the replication analyses for the control-biclusters found in arms 2, 3 and 4.

Dependence of biclustering on minor-allele-frequency

Fig 33 illustrate the relationship between the trace computed using maf$\geq$ 05%, and the trace in the main-text, which was computed using maf$\geq$ 25%. A similar comparision is shown in Fig 34 between a trace computed with a small amount of jitter (in this case eliminating only a single subject or allele-combination per iteration) and the trace in the main-text (which uses an elimination-fraction of $\gamma = 0.5^8 \sim 0.004$).

Illustration of case- and control-subject distribution

Figs 35-42 show heatmaps of the distribution of cases- and control-subjects from arm-2, after projection onto the dominant two principal-components of the bicluster found in arm-1. The different figures correspond to different delineations of the bicluster, as determined by the iteration-index i.

Case-specific biclusters in arms 2, 3 and 4

Figs 43-45 show the traces resulting after we use our biclustering algorithm to search for case-specific biclusters in arms 2-4.

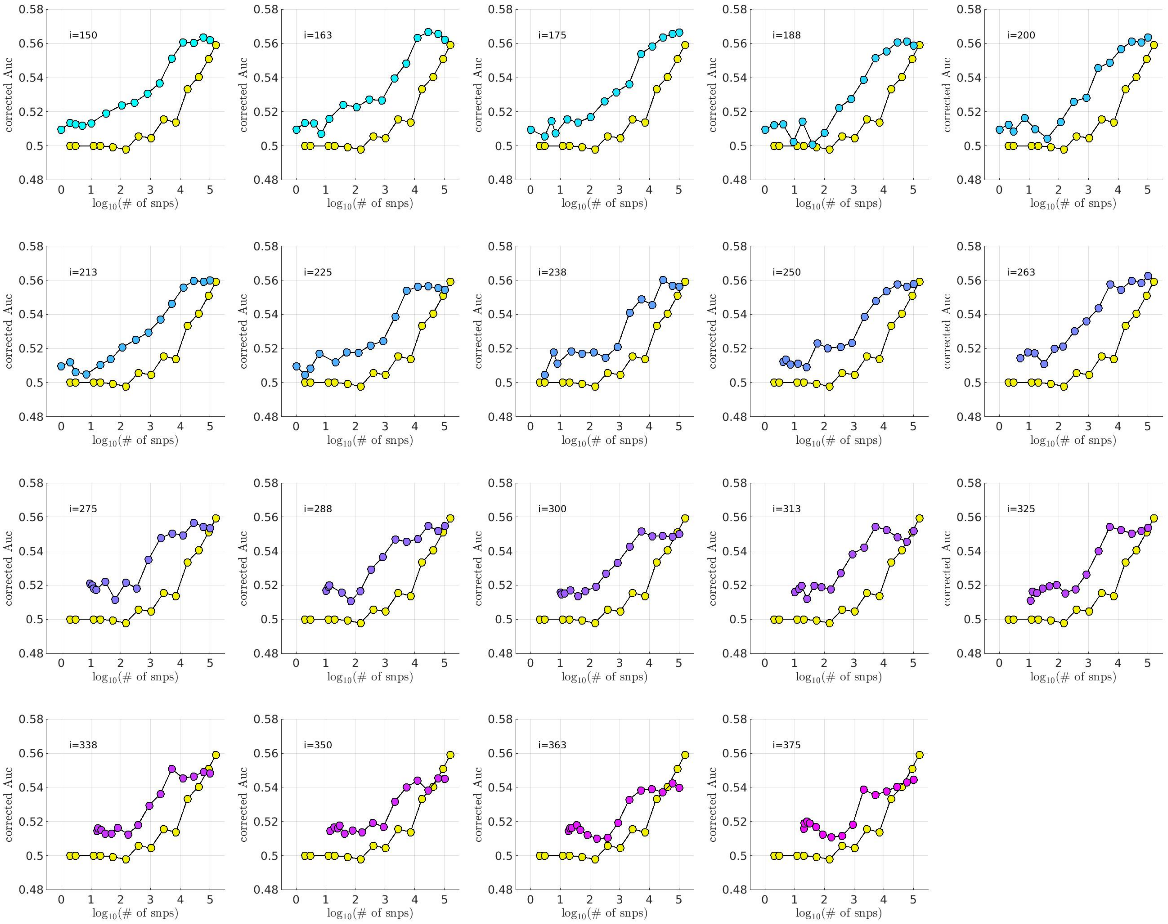

Figure 24: This figure is analogous to Fig 7, except that we test on arm-4 rather than arm-2.

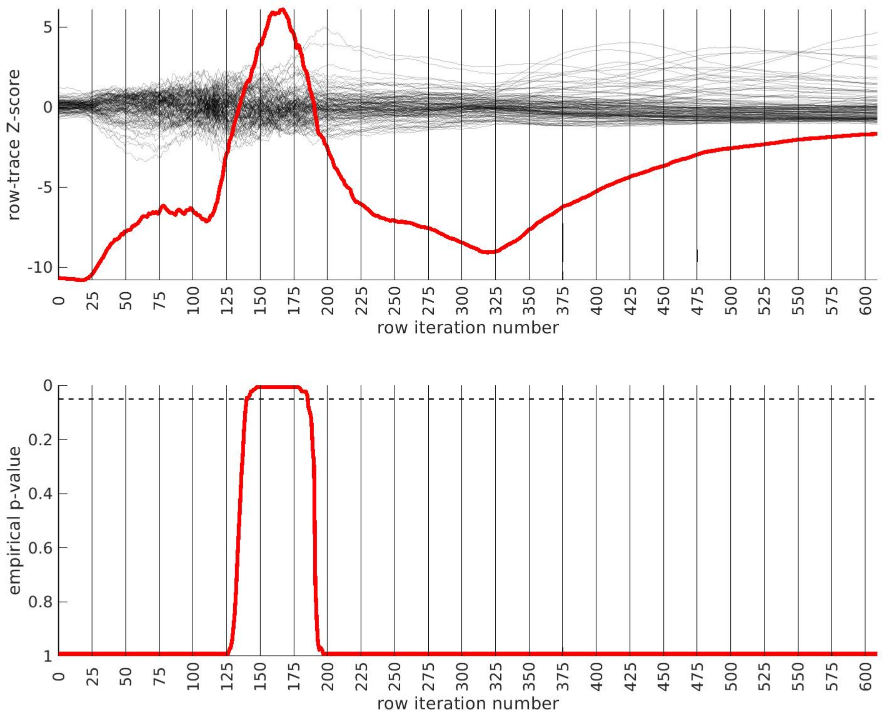

Figure 25: This figure is analogous to Fig 2, except that the red trace corresponds to a search for a secondary bicluster. Note that the red trace is only somewhat significant over a small range of iterations, including $i \in [150, 175]$.

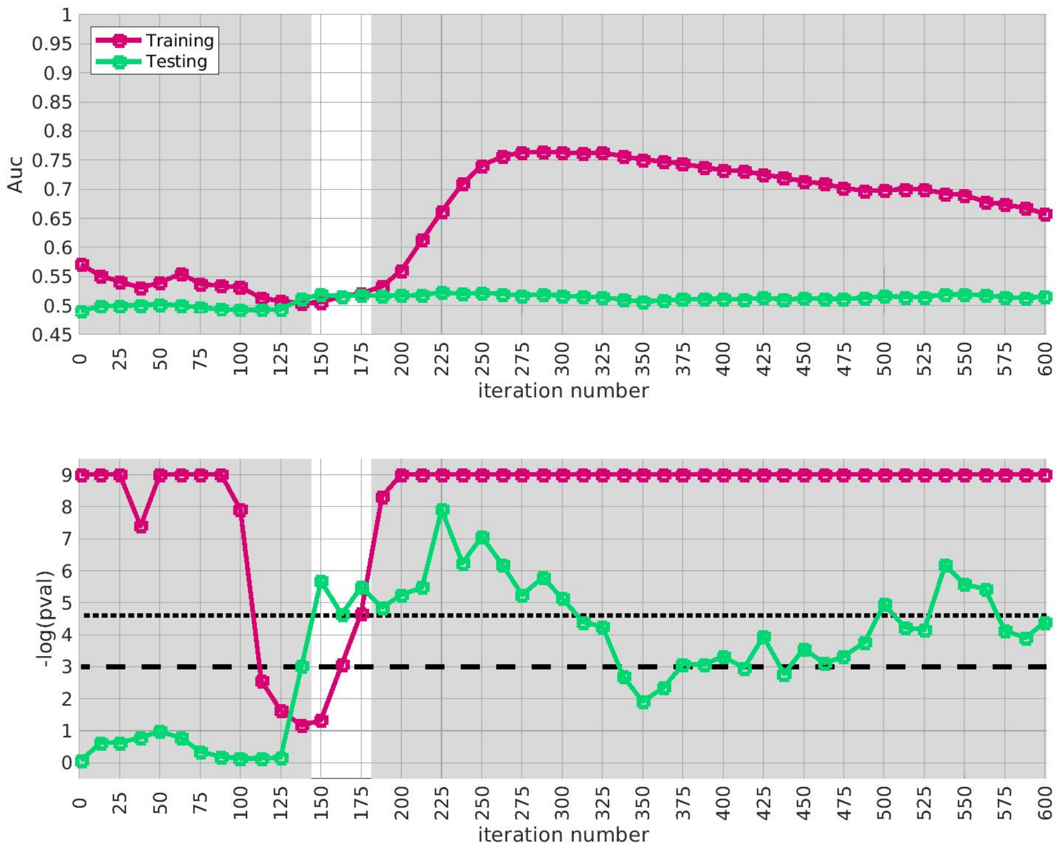

Figure 26: In this figure we illustrate the replication of the secondary bicluster in arm-2. The overall replication p-value is $p = 0.86$ (i.e., not significant).

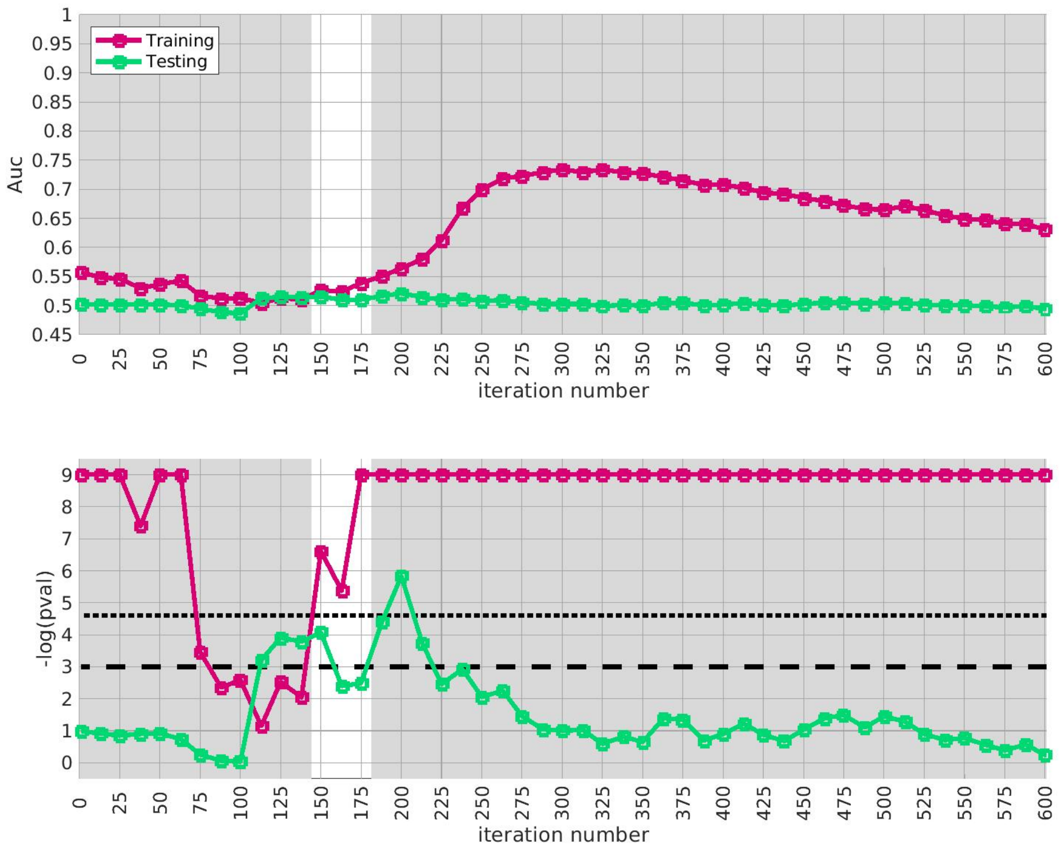

Figure 27: In this figure we illustrate the replication of the secondary bicluster in arm-3. The overall replication p-value is $p = 0.41$ (i.e., not significant).

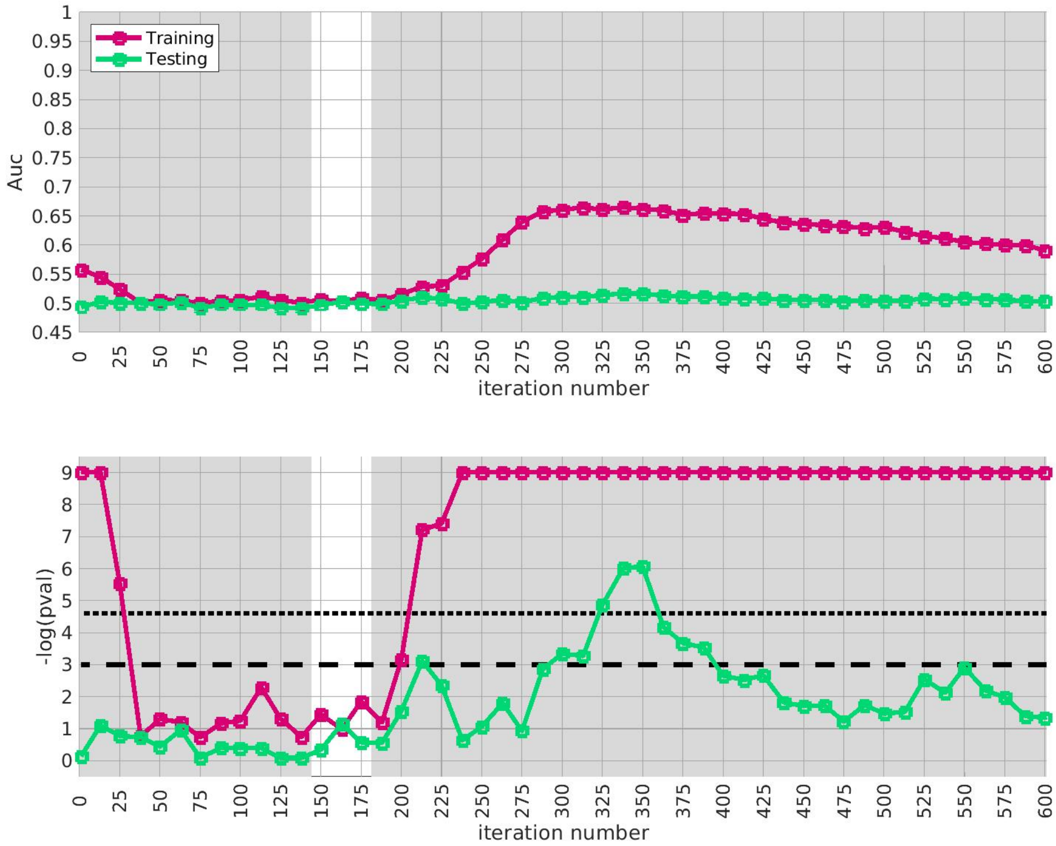

Figure 28: In this figure we illustrate the replication of the secondary bicluster in arm-4. The overall replication p-value is $p = 0.85$ (i.e., not significant).

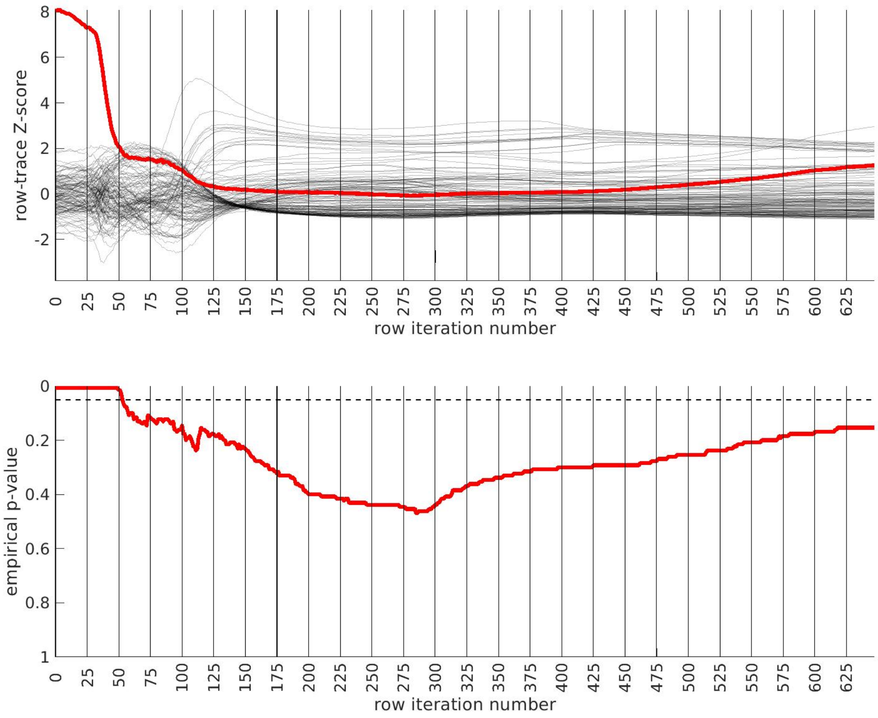

Figure 29: This figure is analogous to Fig 2, except that the red trace corresponds to a search for a bicluster within the control-population of arm-1, rather than the case-population. Note that the red trace decays monotonically, with no distinguished peaks as the algorithm proceeds.

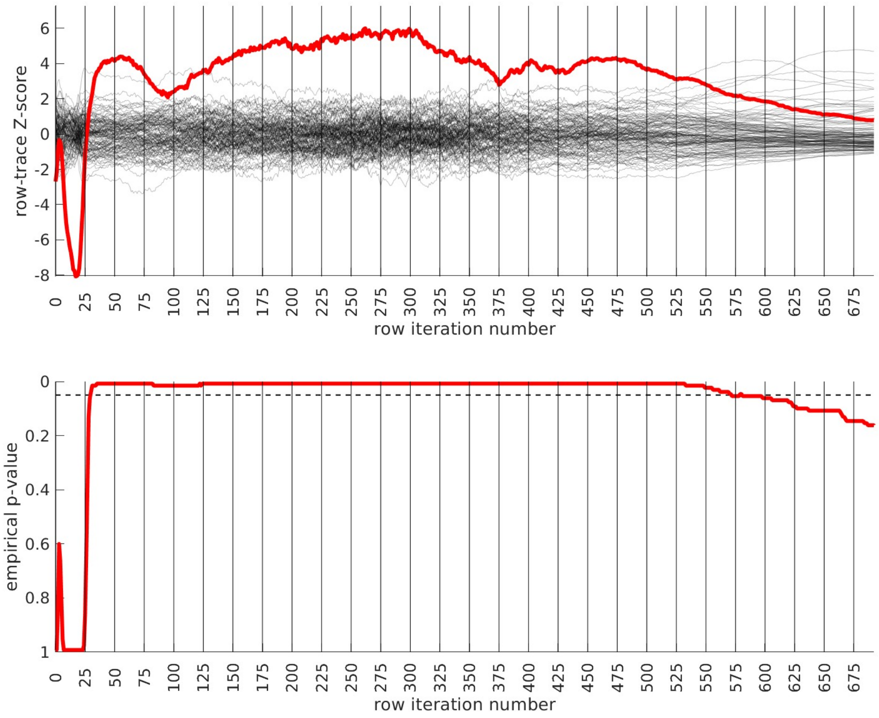

Figure 30: This figure is analogous to Fig 29, except that we consider arm-2 as a training-arm (rather than arm-1). Note that there are multiple distinguished peaks to the red trace, indicating (at least) one bicluster. The overall replication p-value of the dominant bicluster indicated by this trace is $p = 0.030$ in arm-3, $p = 0.17$ in arm-4 and $p = 0.00015$ in arm-1.

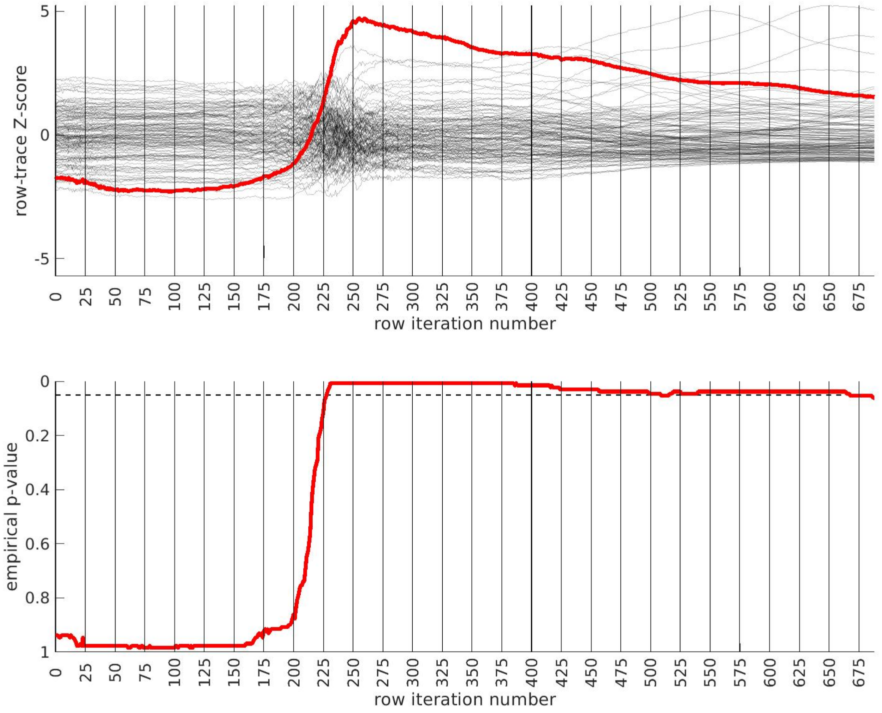

Figure 31: This figure is analogous to Fig 29, except that we consider arm-3 as a training-arm (rather than arm-1). Note that there is a distinguished peak to the red trace, indicating one bicluster. The overall replication p-value of this dominant bicluster is $p = 0.00010$ in arm-2, $p = 0.0025$ in arm-4, and $p = 0.0063$ in arm-1.

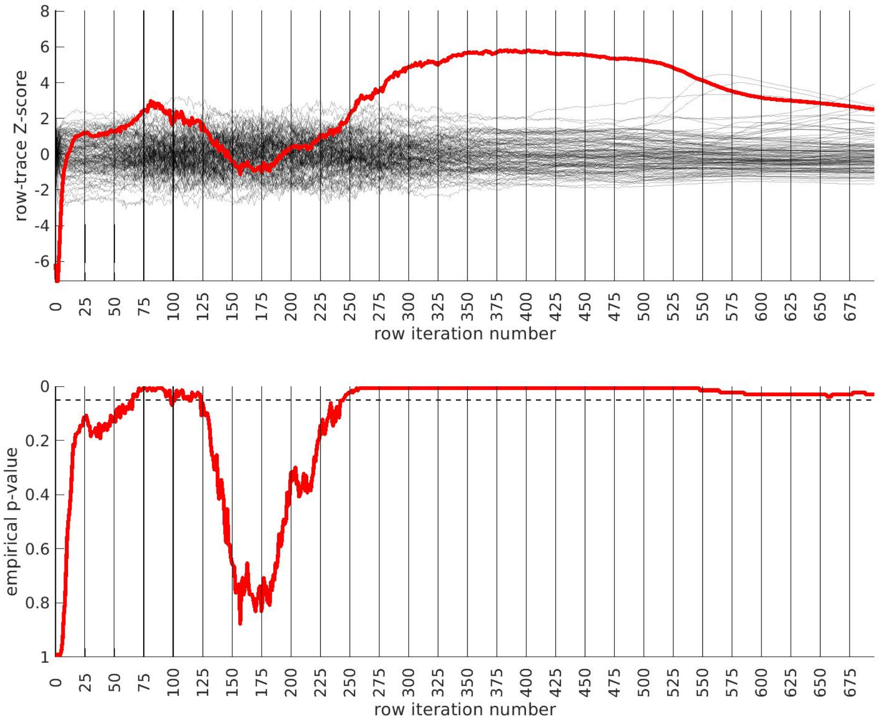

Figure 32: This figure is analogous to Fig 29, except that we consider arm-4 as a training-arm (rather than arm-1). Note that there are multiple distinguished peaks to the red trace, indicating (at least) one bicluster. The overall replication p-value of the dominant bicluster indicated by this trace is $p = 0.000025$ in arm-2, $p = 0.00013$ in arm-3, and $p = 0.040$ in arm-1.

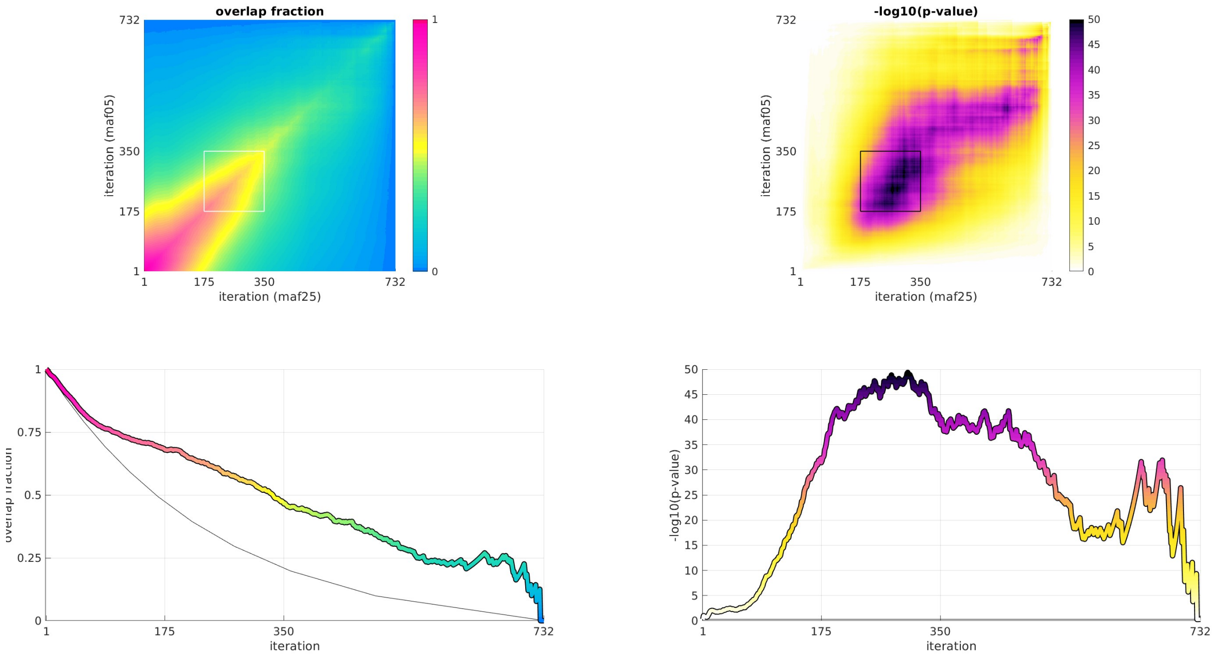

Figure 33: In the main text we used a minor-allele-frequency (i.e., maf) threshold of 0.25 when searching for the bicluster described in Fig 2. If, instead, we use an maf-threshold of 0.05, we also find a significant bicluster, albeit one that includes more allele-combinations and a slightly different selection of case-subjects. Illustrated in this figure is the overlap between these two biclusters. The first panel shows the overlap in case-subjects as a function of iteration. The iterations for maf-threshold 0.25 (i.e., the bicluster described in the main text) are shown along the horizontal, while the vertical axis corresponds to iterations for maf-threshold 0.05. In the lower-left subplot we examine comparable iterations (i.e., involving the same number of case-subjects). Here, we see that the overlap in case-subjects (colored using the colormap in the upper-left) is much higher than chance-level (black). The corresponding p-value for this case-subject overlap is shown in the upper-right. In the lower-right subplot we illustrate the p-value for comparable iterations. Note that the p-value peaks for iterations in the range $[175 - 350]$, which is the range where the original bicluster was most significant. We view this as corroboration that our discovery process (within arm-1) is robust.

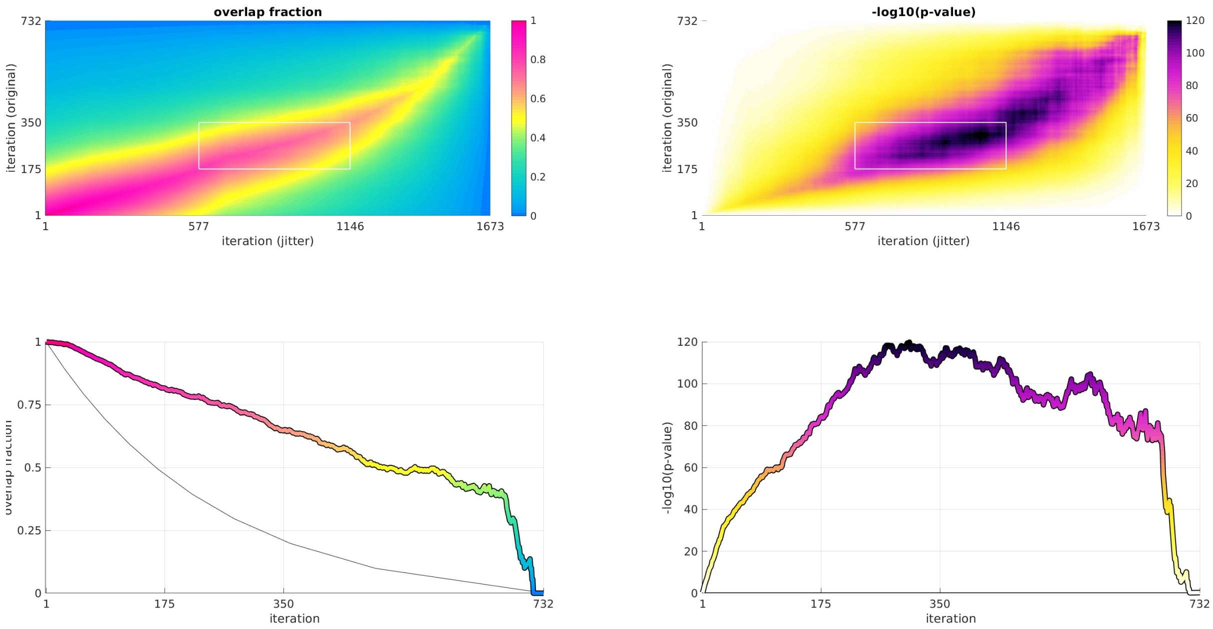

Figure 34: This figure is similar to Fig 33, except that this time we compare the biclustering results from the original biclustering run with a biclustering run where we recalculate the scores after each individual subject and allele-combination is removed (adding a small amount of 'jitter' to the original algorithm). In the upper-left we show the overlap in case-subjects between the jittered algorithm (horizontal) and original algorithm (vertical). The iterations $[175, 350]$ for the original algorithm correspond to iterations $[577, 1146]$ for the jittered algorithm (see highlighted box in upper subplots). The overlap-fraction and p-values for comparable iterations are shown in the bottom subplots. Again, the overlap in case-subjects is very significant, with p-values below 10^{-80} for the range of iterations considered in the main text.

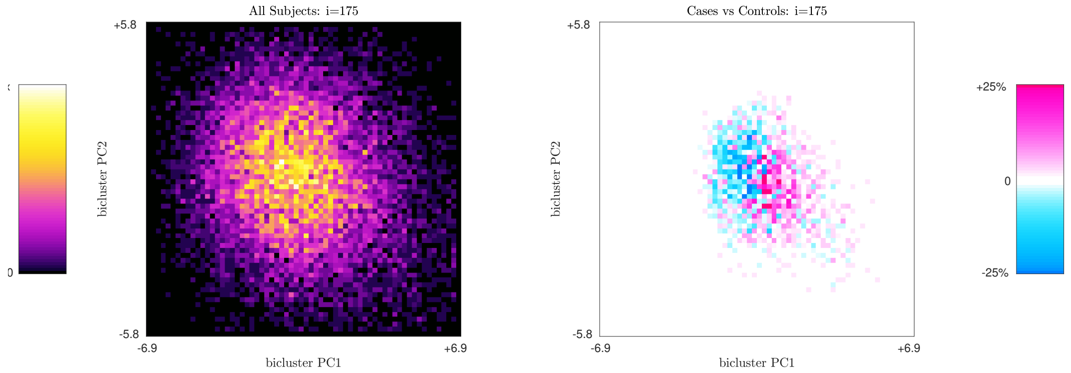

Figure 35: This figure illustrates a 'heatmap' displaying the subjects in arm-2 projected onto the first- and second-principal-components of the bicluster discovered in arm-1 and defined using iteration $i = 175$. The first principal-component (horizontal) is the bicluster-score we use in our replication-study. The second principal-component (vertical) is not used in our analysis. The left subplot shows a heatmap illustrating the distribution of all the subjects in arm-2 (cases and controls). The right subplot shows the difference between the density of cases and controls. The color pink corresponds to areas with a higher case-density than control-density, while blue corresponds to areas with a higher control-density than case-density. The colorbar (far right) ranges across $\pm 25\%$ of the maximum density (taken across both the case- and control-distributions).

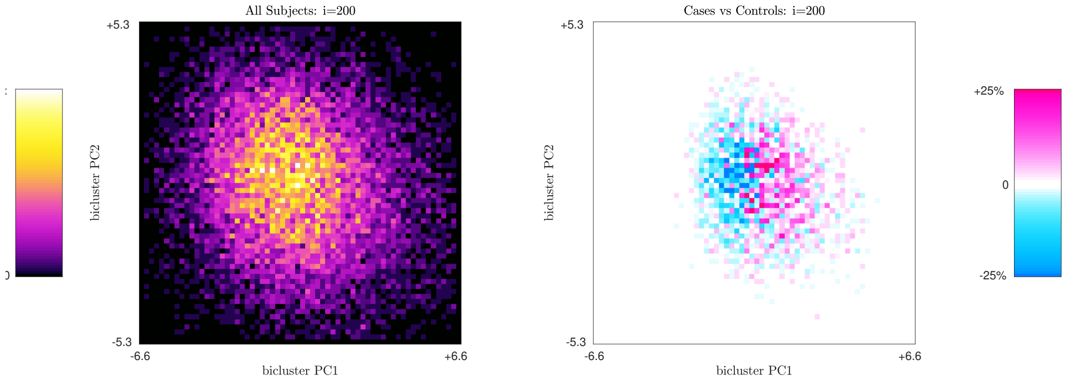

Figure 36: This figure is analogous to Fig 35, except that we use iteration $i = 200$.

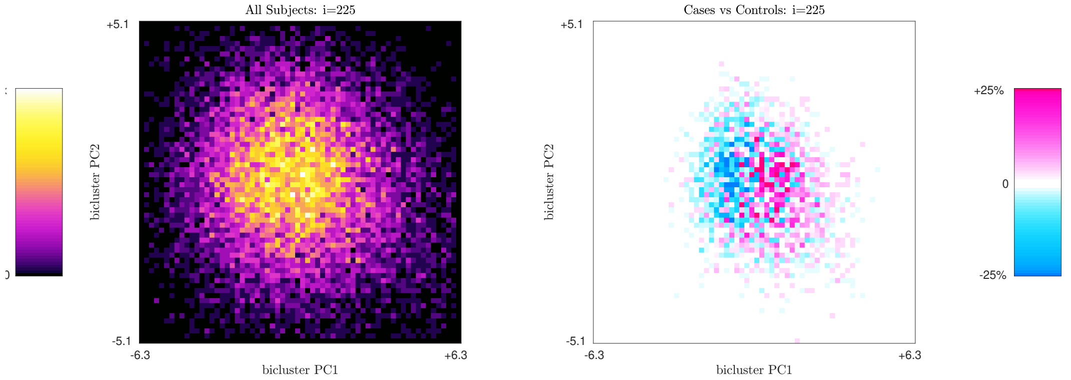

Figure 37: This figure is analogous to Fig 35, except that we use iteration $i = 225$.

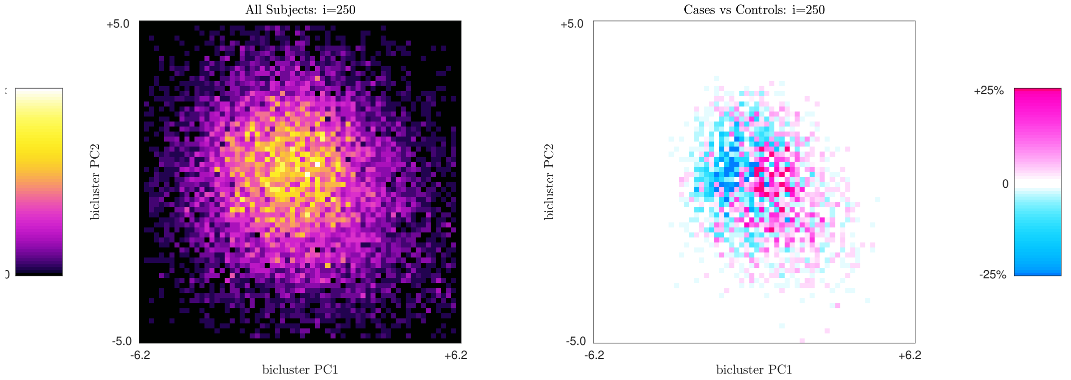

Figure 38: This figure is analogous to Fig 35, except that we use iteration $i = 250$.

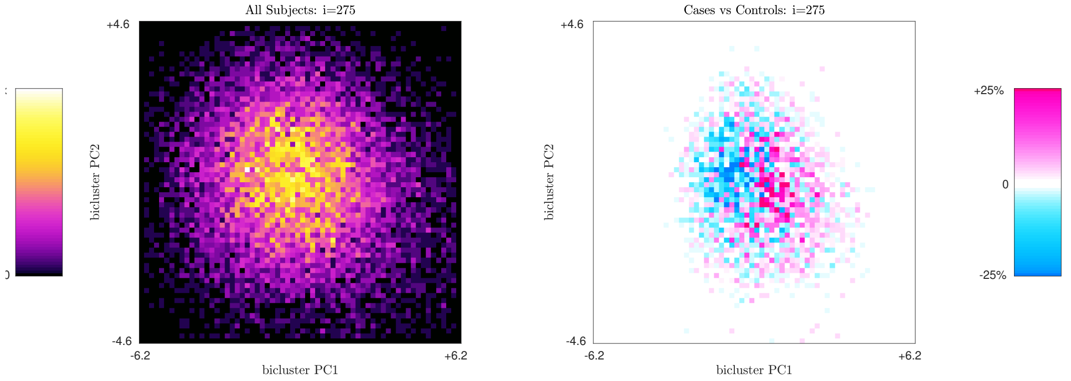

Figure 39: This figure is analogous to Fig 35, except that we use iteration $i = 275$.

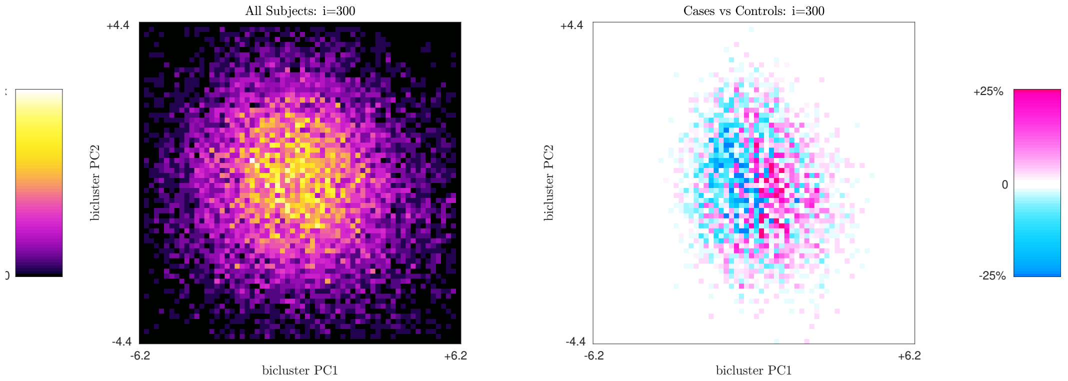

Figure 40: This figure is analogous to Fig 35, except that we use iteration $i = 300$.

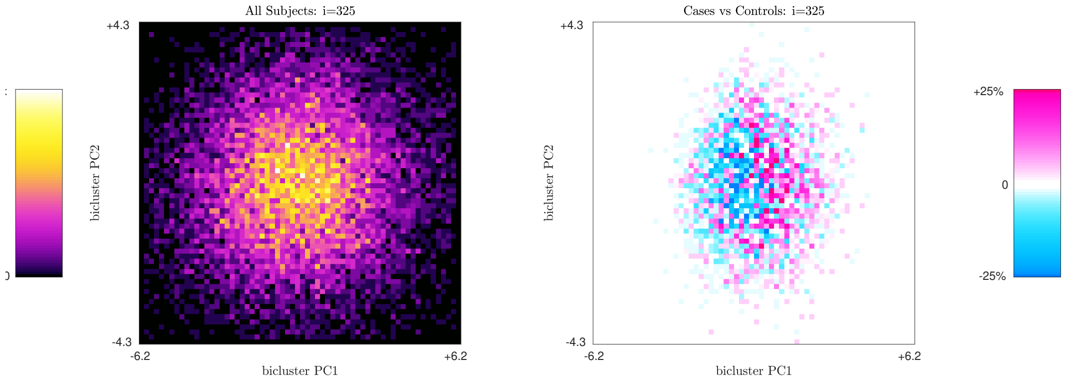

Figure 41: This figure is analogous to Fig 35, except that we use iteration $i = 325$.

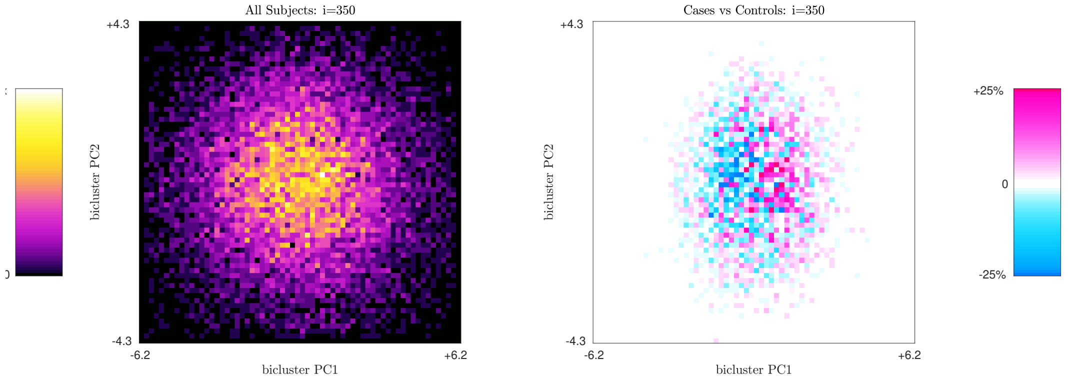

Figure 42: This figure is analogous to Fig 35, except that we use iteration $i = 350$.

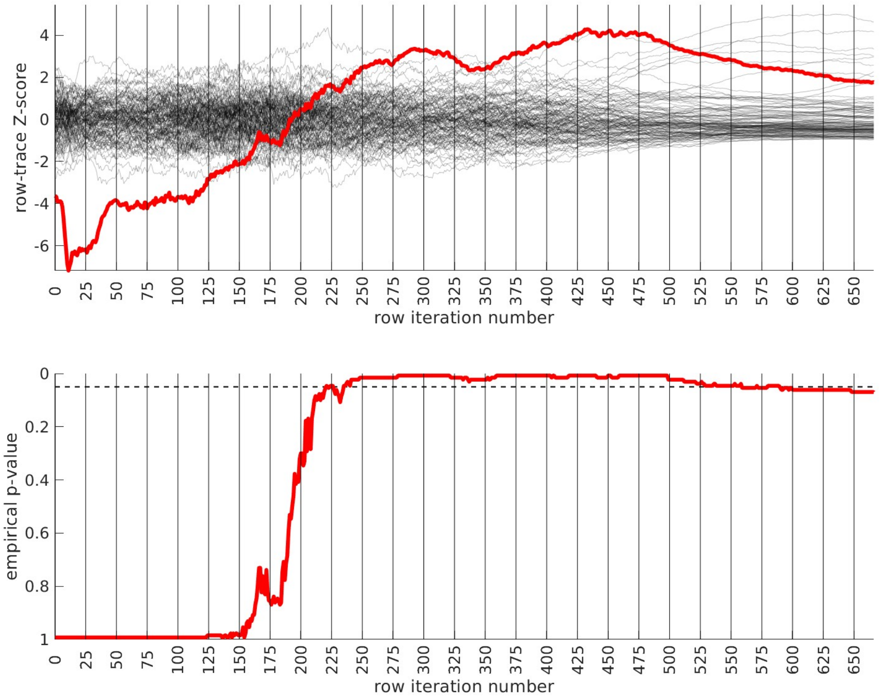

Figure 43: This figure is analogous to Fig 2, except that we show the traces resulting after we run our biclustering algorithm to search for case-specific biclusters using arm-2 as the discovery arm (again limited to SNPs with maf ≥ 0.25). The overall p-value for the data (red-trace), estimated using the strategy in [65], is $p \sim 6.5/125 \sim 0.051$.

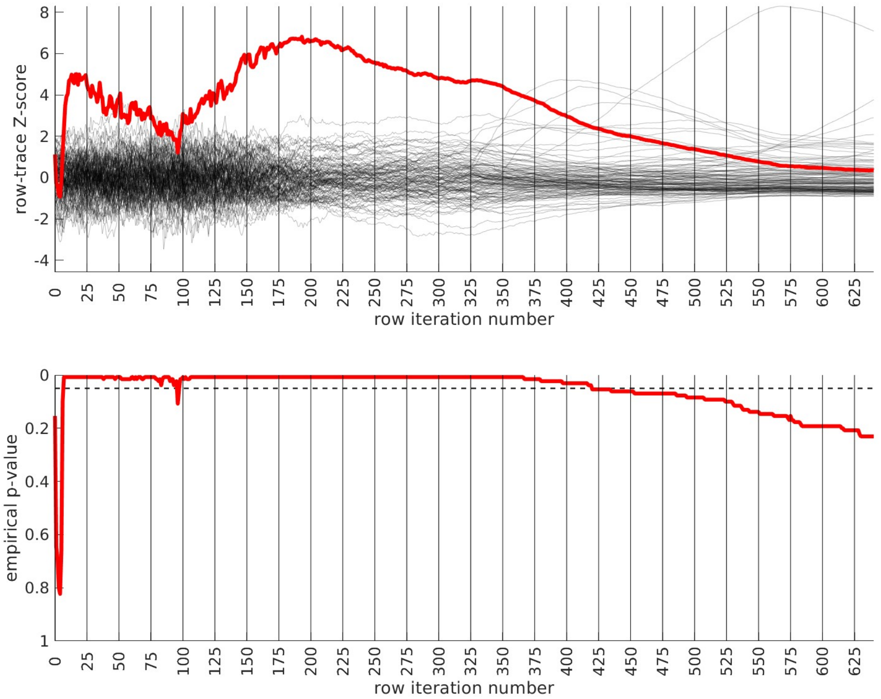

Figure 44: This figure is analogous to Figs 2 and 43, except that we show the traces resulting after we run our biclustering algorithm using arm-3 as the discovery arm. The overall p-value for the data is $p \lesssim 1/64$.

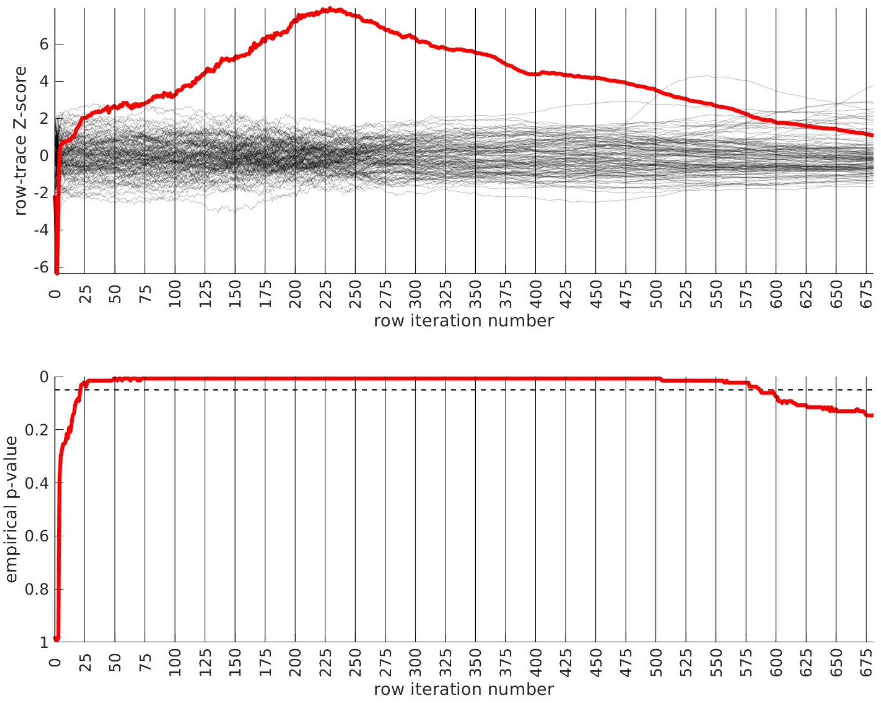

Figure 45: This figure is analogous to Figs 2, 43 and 44, except that we use arm-3 as the discovery arm. The overall p-value for the data is $p \lesssim 1/128$.
