# Supplementary material for: Heterogeneity analysis provides evidence for a genetically homogeneous subtype of bipolar-disorder
Source: ArXiv. 2024 Apr 30:arXiv:2405.00159v1. Preprint. [Version 1] (PMC11092873)
Supplement: Supplementary file 1 [file NIHPP2405.00159V1-supplement-1.pdf]

Supporting information

Additional replication analyses in arm-2

Figs 11 and 12 illustrate the replication obsreved in arm-2 when the SNP-overlap between arm-1 and arm-2 is artificially decreased.

Influence of covariates on the bicluster

Figs 13 and 14 illustrate the association between the bicluster found in arm-1 and the ancestry-related covariates. Fig. 15 illustrates the association with sex.

Interaction between bicluster-score and population-wide PRS

As described in the Methods section, we calculated the population-wide $\mathbf{PRS}_{\text{wide}}(j';\tilde{p})$ and the bicluster-informed $\mathbf{PRS}_{\text{bicl}}(j';i,\tilde{p})$ across a variety of iterations i and $\tilde{p}$-thresholds. In Fig 16 we illustrate the correspondence between the population-wide $\mathbf{PRS}_{\text{wide}}(j';\tilde{p})$ and bicluster-score $u'_{j'}(i)$ for arm-2 at $i = 175$. This trend persists for other iterations, as illustrated in Figs 17 and 18.

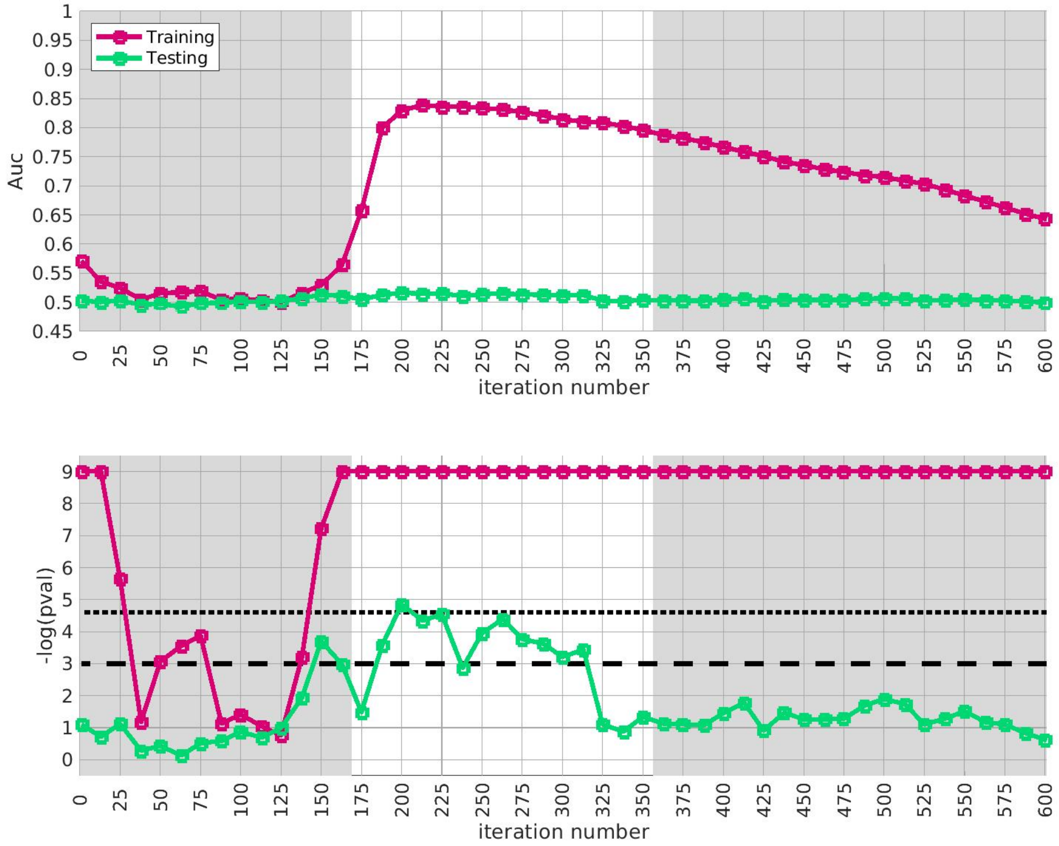

Fig 12. This figure is similar to Fig 3, except that we randomly eliminate SNPs from arm-2 until the SNP-overlap between arm-2 and the training-arm is equal to the SNP-overlap between arm-4 and the training-arm.

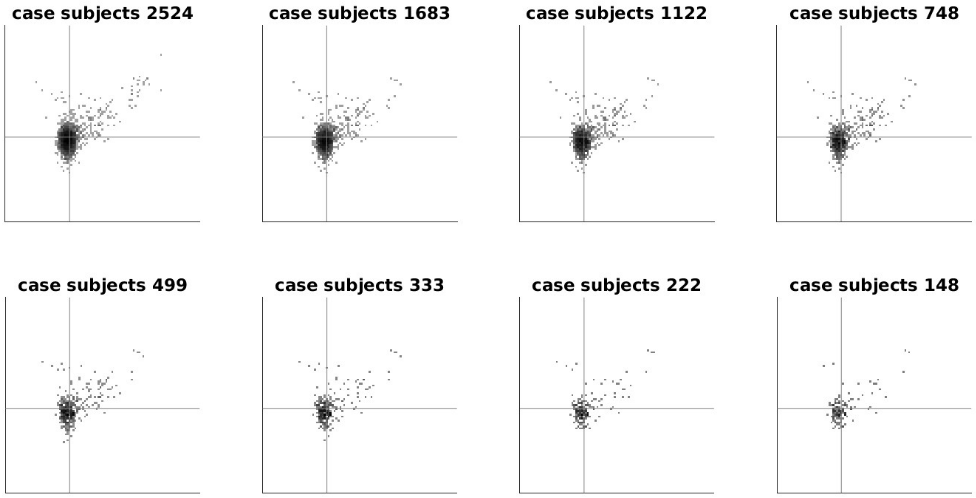

Fig 13. This figure illustrates the distribution of ancestry-associated principal-components U_1^1 and U_2^1 amongst the remaining case-subjects as the half-loop algorithm proceeds (specifically, at iterations $i \in \{96, 189, 276, 359, 442, 511, 585\}$). Each subplot displays a scatterplot of remaining case-subjects plotted with respect to U_1^1 and U_2^1. The number of remaining case-subjects is shown above each subplot. Note that the overall shape of the distribution does not change much as the algorithm proceeds.

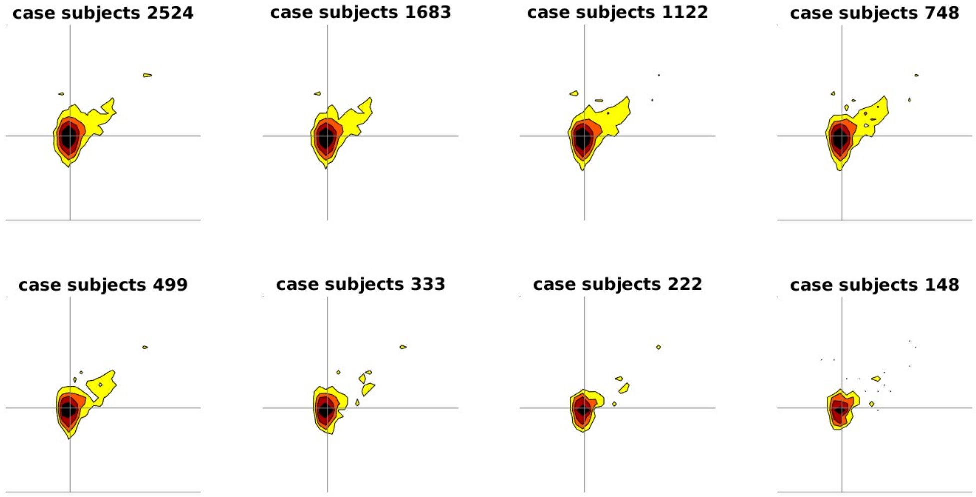

Fig 14. This figure is similar to Fig 13, except that a heat-map of the distribution is shown (rather than a scatterplot). The colors in the heat-map correspond to the logarithm of the density in the underlying distribution. Four different contours are shown, ranging from yellow to maroon, corresponding to the 20%, 40%, 60% and 80% percentiles of the log-density.

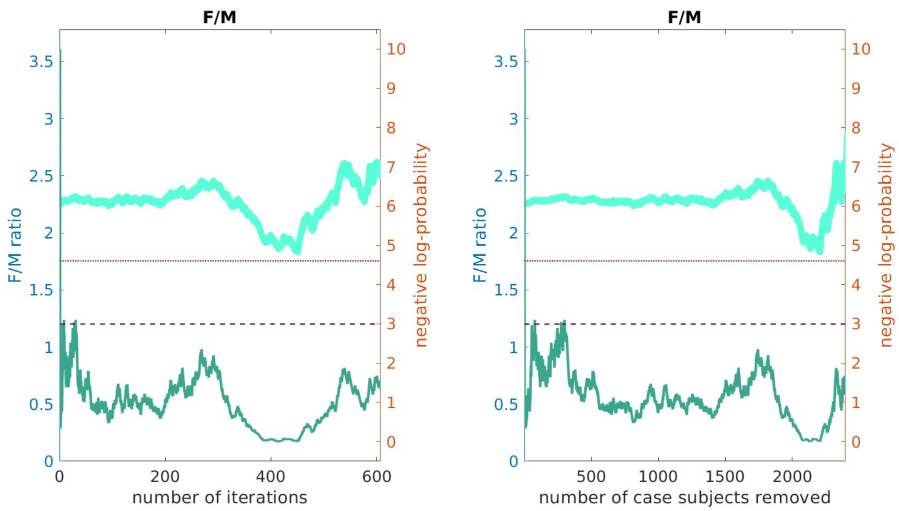

Fig 15. This figure plots the ratio of female to male subjects within $\mathcal{J}(i)$ (light-teal, left y-axis) as a function of the iteration i (left) and the number of remaining case-subjects (right). The dark-teal line corresponds to the negative-log-probability (right y-axis) of observing a ratio at least as large by chance. The dashed and dotted horizontal lines indicate 0.05 and 0.01 significance values, respectively. Note that the female population is not over-represented across the range of iterations including $i \in [175, 350]$, implying that the bicluster we observe is not significantly enriched for female subjects.

Nagelkerke R^2 for a series of linear-models

To complement the observations of Figs 16, 17 and 18, we investigate how the bicluster-informed PRSs and the bicluster-scores themselves contribute to risk-prediction. For a given i and $\tilde{p}$ we build a series of linear-models to predict case-control status. These linear-models use: (i) the ancestry-related covariates, (ii) the population-wide $\mathbf{PRS}_{\text{wide}}(j'; \tilde{p})$, (iii) the bicluster-informed $\mathbf{PRS}_{\text{bicl}}(j'; i, \tilde{p})$, and (iv) the bicluster-score $u'_{j'}(i)$.

The series of linear-models we consider involve successively more and more of these terms. Thus, the null linear-model attempts to predict case-control status using no terms at all (i.e., using only the average prevalence of cases and controls). The first linear-model attempts to predict case-control status using only term (i), that is, only the ancestry-related covariates. The second linear-model uses both term (i) and (ii), i.e., both the ancestry-related covariates and the population-wide PRS. The third linear-model uses terms (i), (ii) and (iii). And finally, the fourth linear-model uses terms (i)-(iv). For each linear-model we measure the Nagelkerke pseudo-r^2 value between that model and the null model The difference in pseudo-r^2 values gives an estimate of the additional explanatory power provided by each term in succession.

Results for various i and $\tilde{p}$ are shown in Figs 19 and 20. Each vertical bar in this figure corresponds to a particular i and $\tilde{p}$. Each vertical bar is further divided into segments illustrating the incremental Nagelkerke pseudo-r^2 value associated with each term. Note that the bicluster-informed PRS, as well as the bicluster-score $u'_{j'}(i)$ add explanatory power to the underlying linear model. This phenomena is most pronounced when i is in the middle of the range $i \in [175, 350]$ and the threshold $\tilde{p}$ is small.

The observations in Figs 16-20 suggest that the bicluster might contain information useful for improving risk-prediction. Moroever, the peak in overall R^2 when $\tilde{p} \sim 1e-2$ suggests that the signal in our bicluster-score involves subsets of SNPs which do not

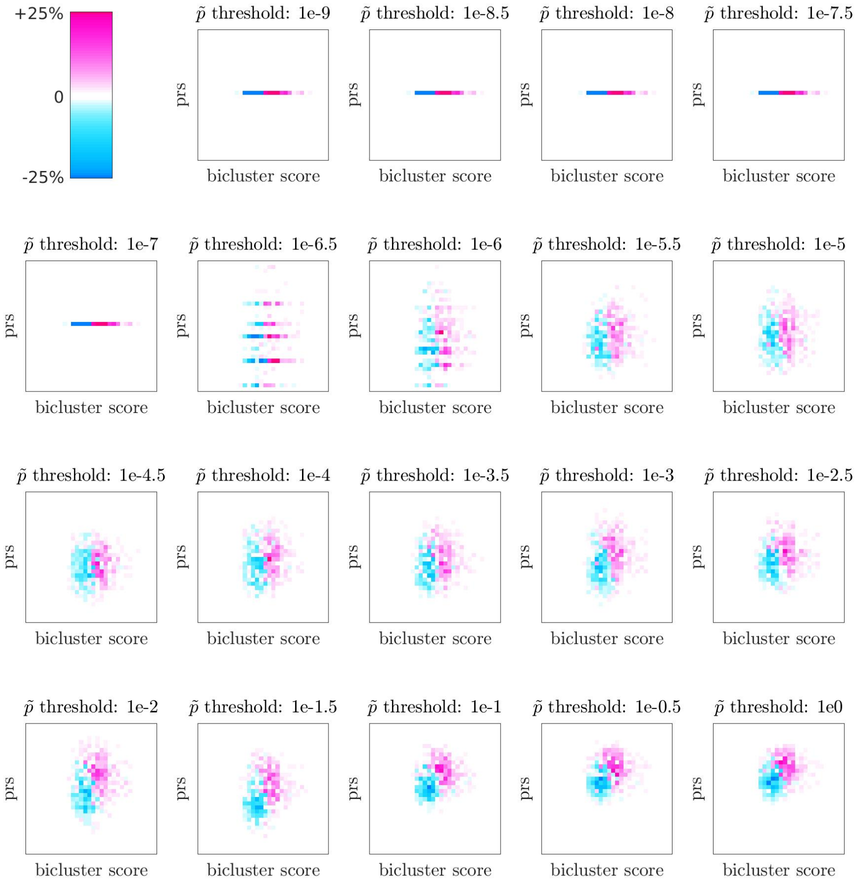

Fig 16. In this figure we illustrate the correspondence between the population-wide $\mathbf{PRS}(j';\tilde{p})$ and bicluster-score $u'_{j'}(i)$ for arm-2 at $i = 175$. Each subplot visualizes the distribution of subjects in arm-2 as a function of bicluster-score $u'_{j'}(i)$ (horizontal) and $\mathbf{PRS}_{\text{wide}}(j';\tilde{p})$ (vertical), with the SNP-p-value threshold $\tilde{p}$ varying across the subplots. In each subplot a heatmap is shown, representing the difference between the density of cases and controls. The color pink corresponds to areas with a higher case-density than control-density, while blue corresponds to areas with a higher control-density than case-density. The colorbar (upper-left) ranges across $\pm 25\%$ of the maximum density (taken across both the case- and control-distributions). Note that, while the bicluster-score is correlated with the population-wide PRS when $\tilde{p}$ is sufficiently high, the correlation is far from perfect. Note that, when $\tilde{p}$ is high (i.e., $\tilde{p} \sim 1$ and all the SNPs are used to generate the PRS), there is a marked correlation between case-control status and high-values of $\mathbf{PRS}_{\text{wide}}(j';\tilde{p})$ and bicluster-score $u'_{j'}(i)$. However, for lower values of $\tilde{p}$ this structure shifts, and $\mathbf{PRS}_{\text{wide}}(j';\tilde{p})$ is no longer a useful indicator of case-control status while the bicluster-score $u'_{j'}(i)$ is still a useful indicator. This trend persists for other iterations, as illustrated in Figs 17 and 18.

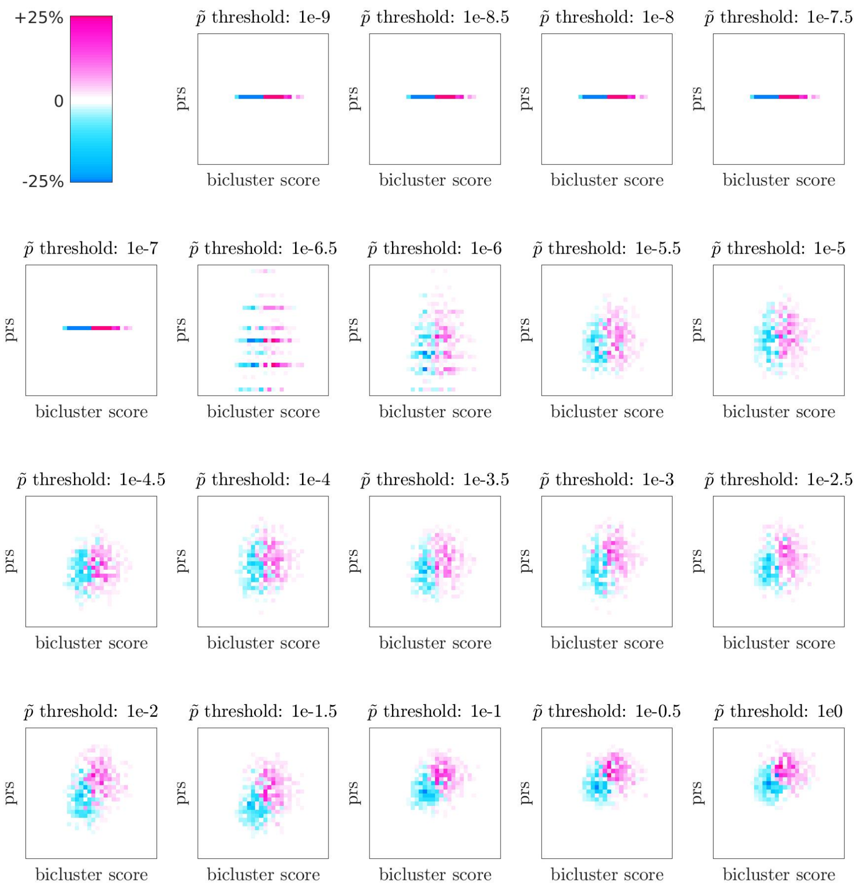

Fig 17. This figure is analogous to Fig 16, except for $i = 225$.

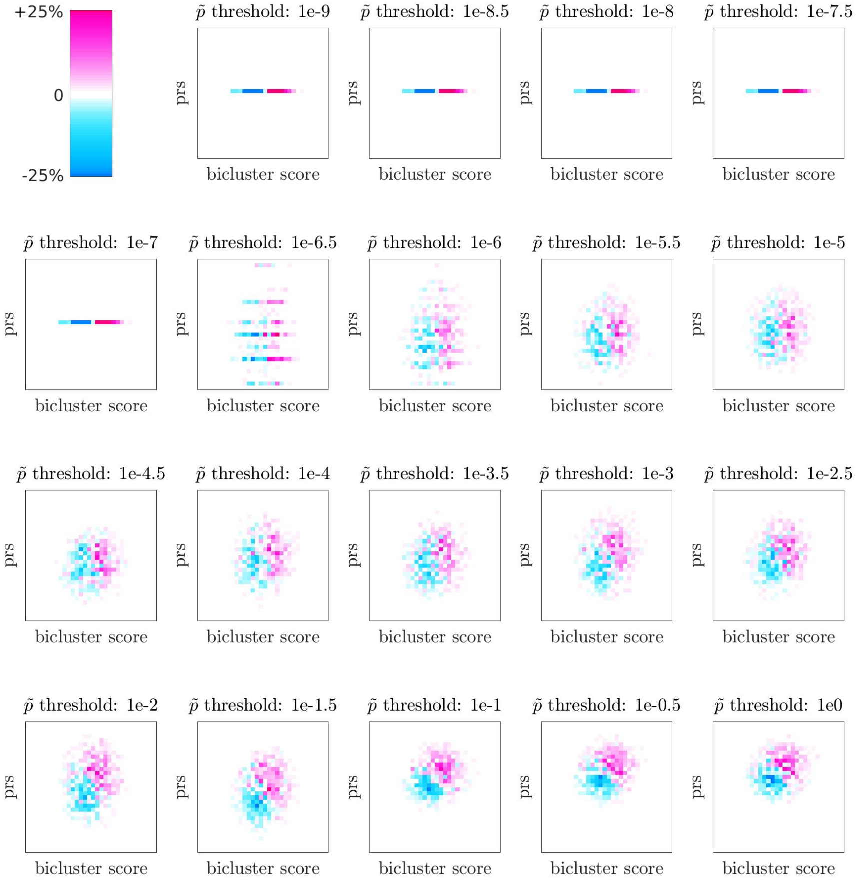

Fig 18. This figure is analogous to Fig 16, except for $i = 350$.

individually achieve genome-wide significance.

Replication results for arm-3 and arm-4

Figs 21 and 22 illustrate the replication analyses on arms 3 and 4.

Significance of secondary bicluster in arm-1

Figs 23 24 25 and 26 illustrate the significance of the secondary bicluster found in arm-1.

Control-biclusters

Figs 27, 28, 29 and 30 illustrate the replication analyses for the control-biclusters found in arms 2, 3 and 4.

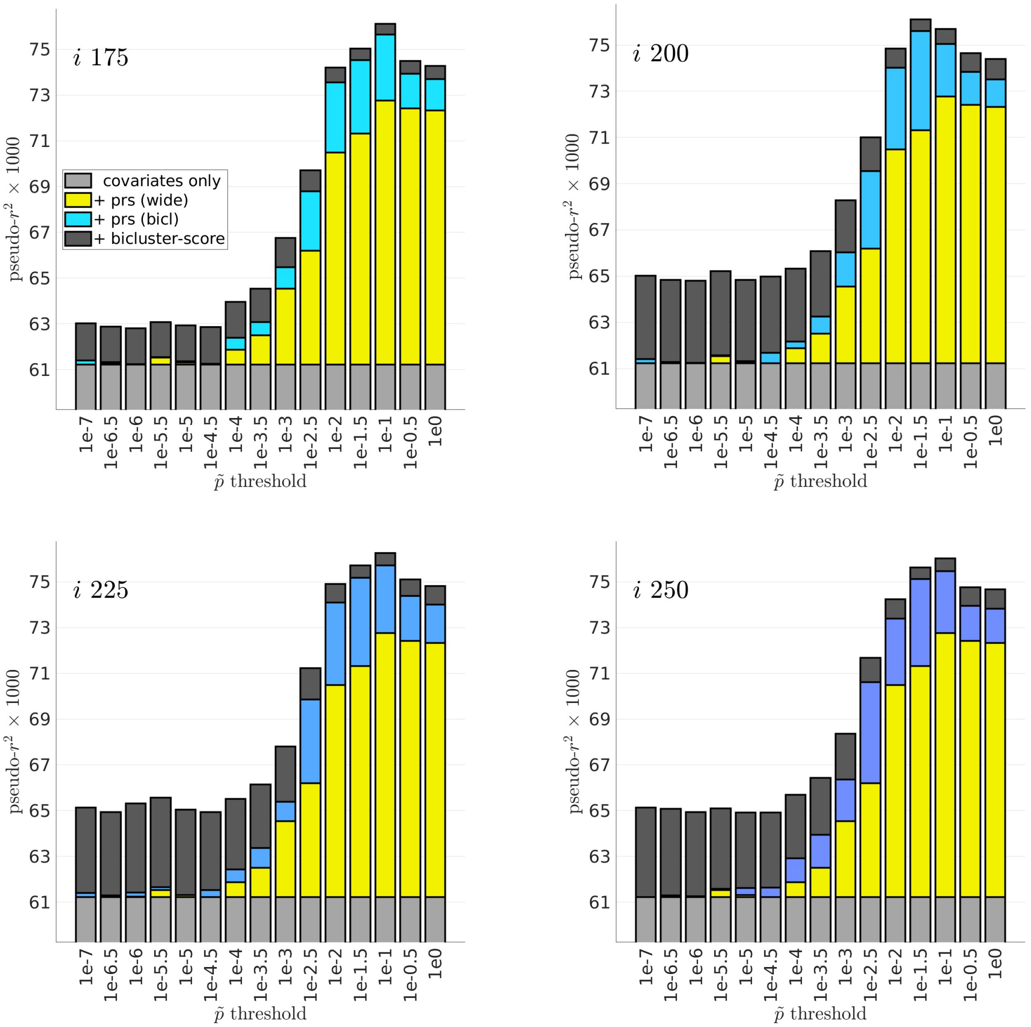

Fig 19. In this figure we illustrate the explanatory power of the bicluster-informed $\mathbf{PRS}_{\text{bicl}}(j'; i, \tilde{p})$ in combination with the bicluster-score $u'_{j'}(i)$ for arm-2, with the iteration number i varying across the subplots. Within each subplot the $\tilde{p}$-threshold is shown along the horizontal-axis. We measure the incremental Nagelkerke pseudo-r^2 value associated with the following terms: (i) the ancestry-related covariates, (ii) the population-wide $\mathbf{PRS}_{\text{wide}}(j'; \tilde{p})$, (iii) the bicluster-informed $\mathbf{PRS}_{\text{bicl}}(j'; i, \tilde{p})$, and (iv) the bicluster-score $u'_{j'}(i)$. For each i and $\tilde{p}$ we show a vertical bar divided into segments illustrating the contribution of each term (i)-(iv), with colors light-grey, yellow, blue-pink and dark-grey (respectively). The colors used for the third term (ranging from blue to pink) correspond to the colors used in Fig 7 below.

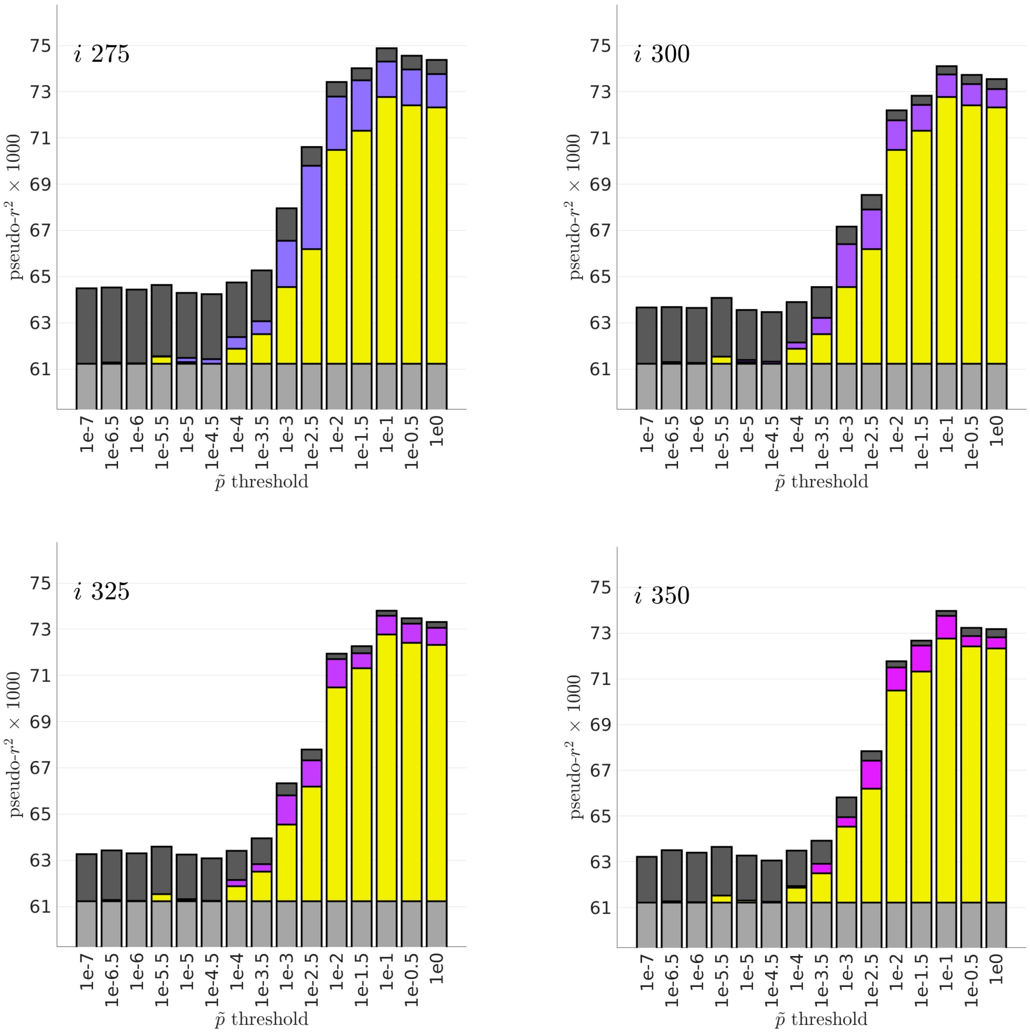

Fig 20. This is analogous to Fig 19, for a different set of iterations.

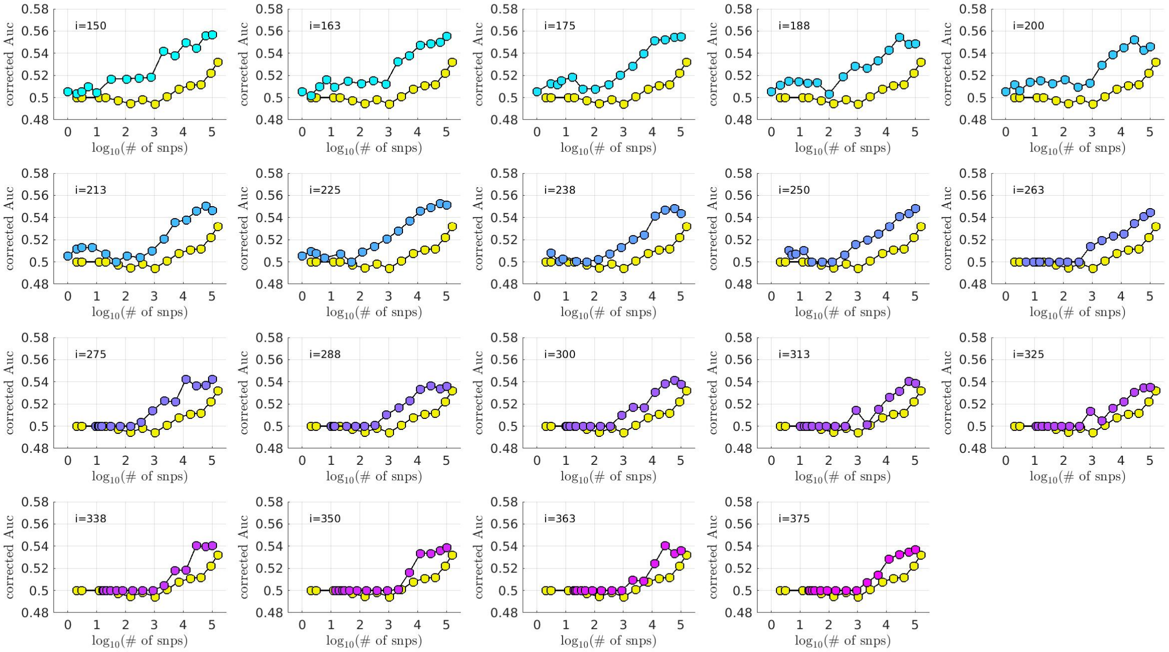

Fig 21. This figure is analogous to Fig 7, except that we test on arm-3 rather than arm-2.

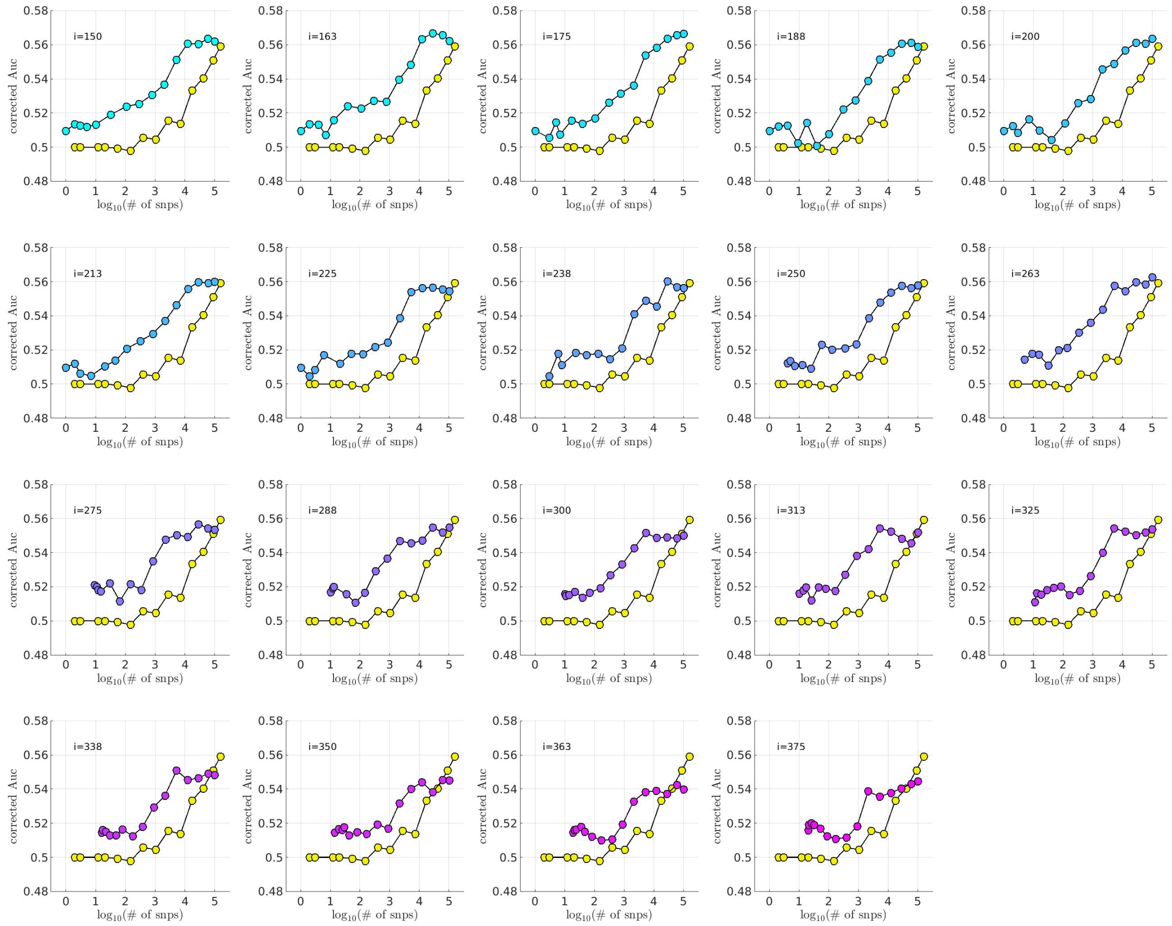

Fig 22. This figure is analogous to Fig 7, except that we test on arm-4 rather than arm-2.

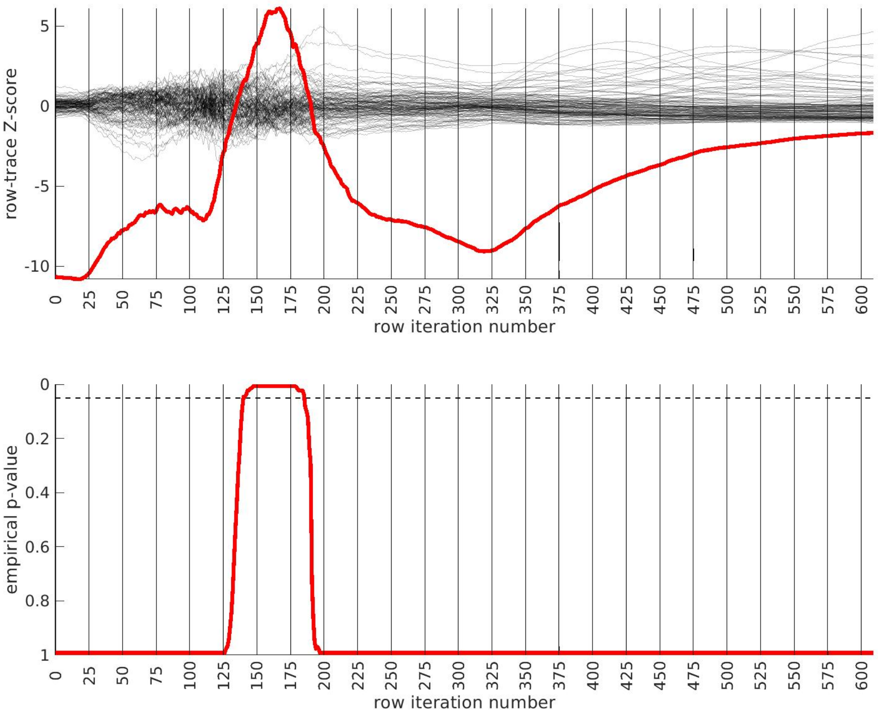

Fig 23. This figure is analogous to Fig 2, except that the red trace corresponds to a search for a secondary bicluster. Note that the red trace is only somewhat significant over a small range of iterations, including $i \in [150, 175]$.

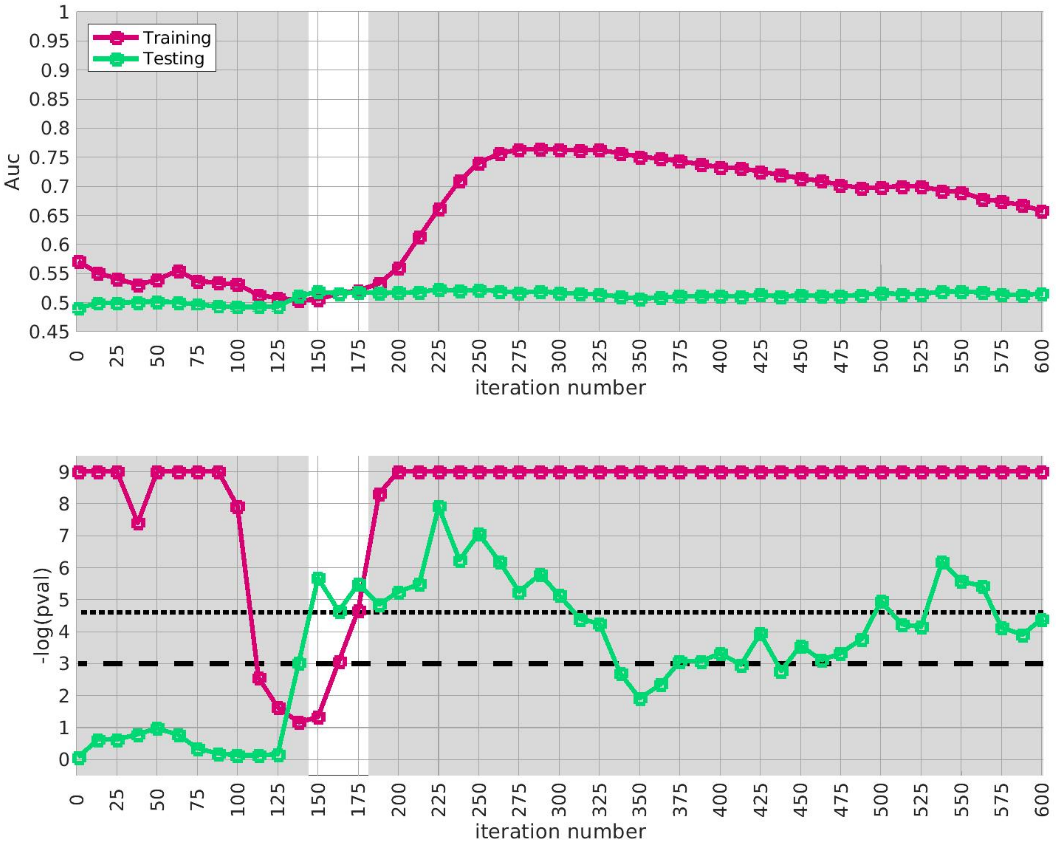

Fig 24. In this figure we illustrate the replication of the secondary bicluster in arm-2. The overall replication p-value is $p = 0.86$ (i.e., not significant).

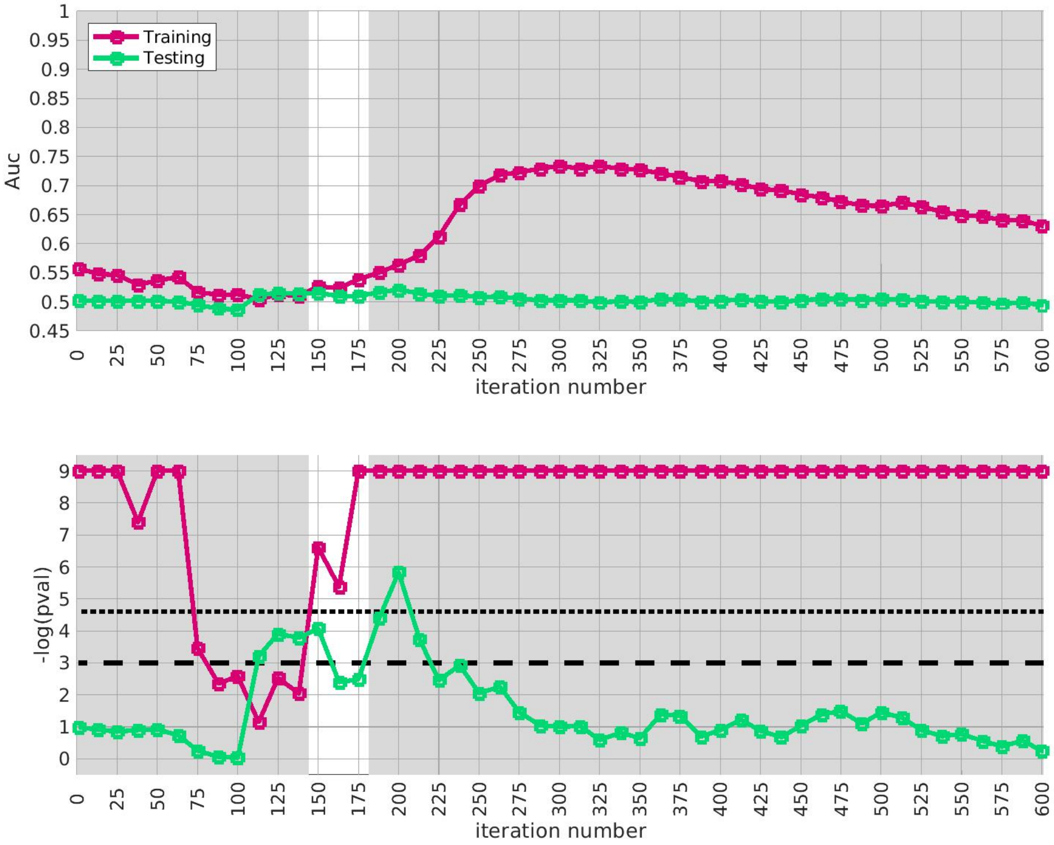

Fig 25. In this figure we illustrate the replication of the secondary bicluster in arm-3. The overall replication p-value is $p = 0.41$ (i.e., not significant).

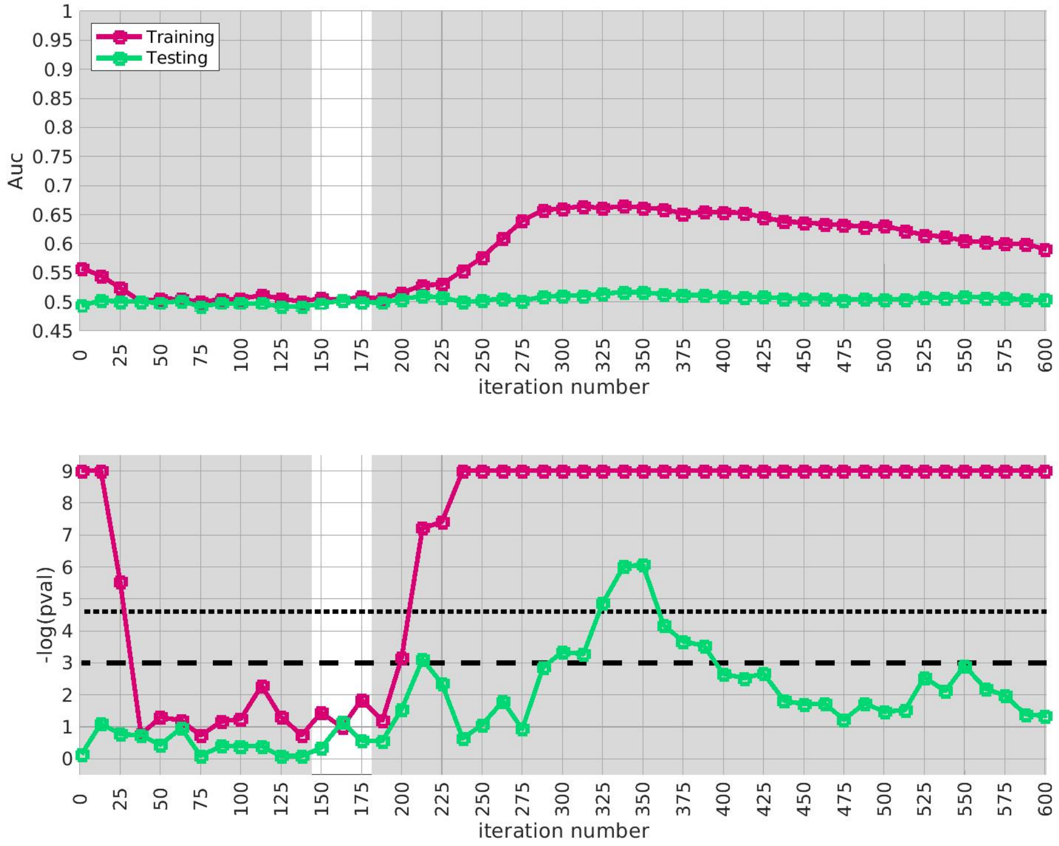

Fig 26. In this figure we illustrate the replication of the secondary bicluster in arm-4. The overall replication p-value is $p = 0.85$ (i.e., not significant).

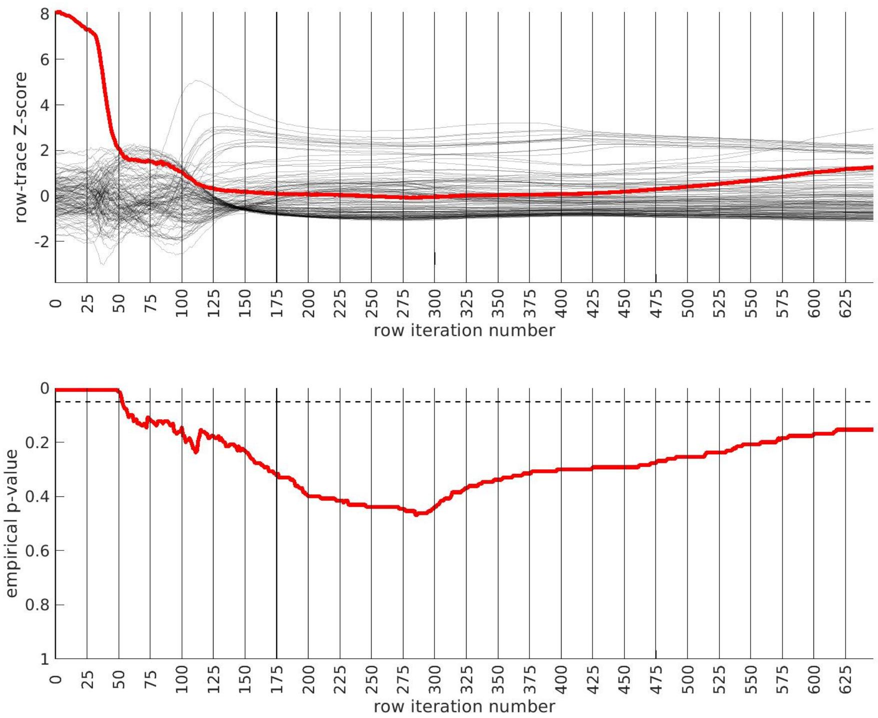

Fig 27. This figure is analogous to Fig 2, except that the red trace corresponds to a search for a bicluster within the control-population of arm-1, rather than the case-population. Note that the red trace decays monotonically, with no distinguished peaks as the algorithm proceeds.

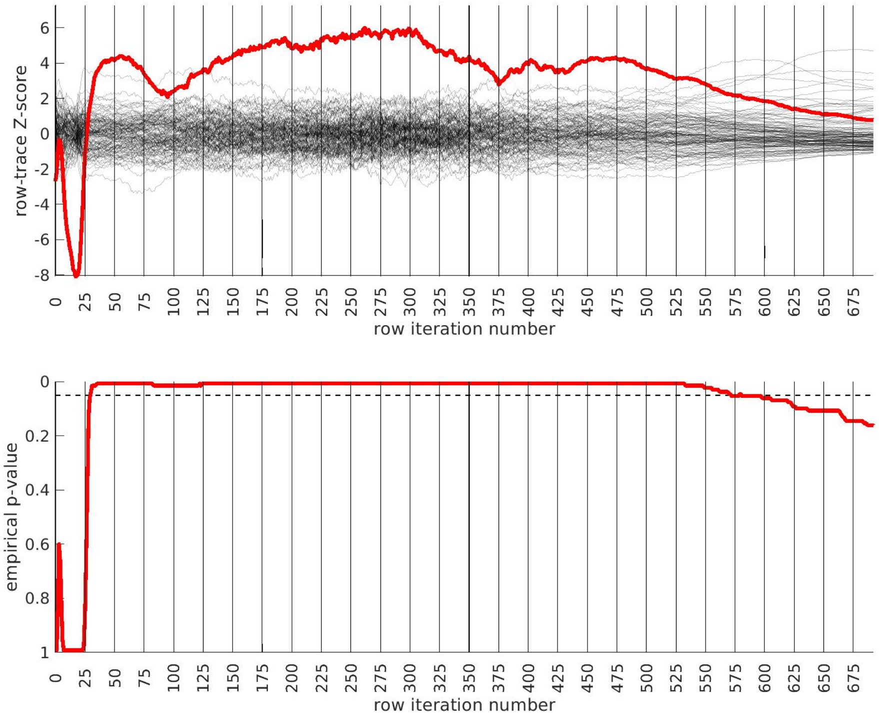

Fig 28. This figure is analogous to Fig 27, except that we consider arm-2 as a training-arm (rather than arm-1). Note that there are multiple distinguished peaks to the red trace, indicating (at least) one bicluster. The overall replication p-value of the dominant bicluster indicated by this trace is $p = 0.030$ in arm-3, $p = 0.17$ in arm-4 and $p = 0.00015$ in arm-1.

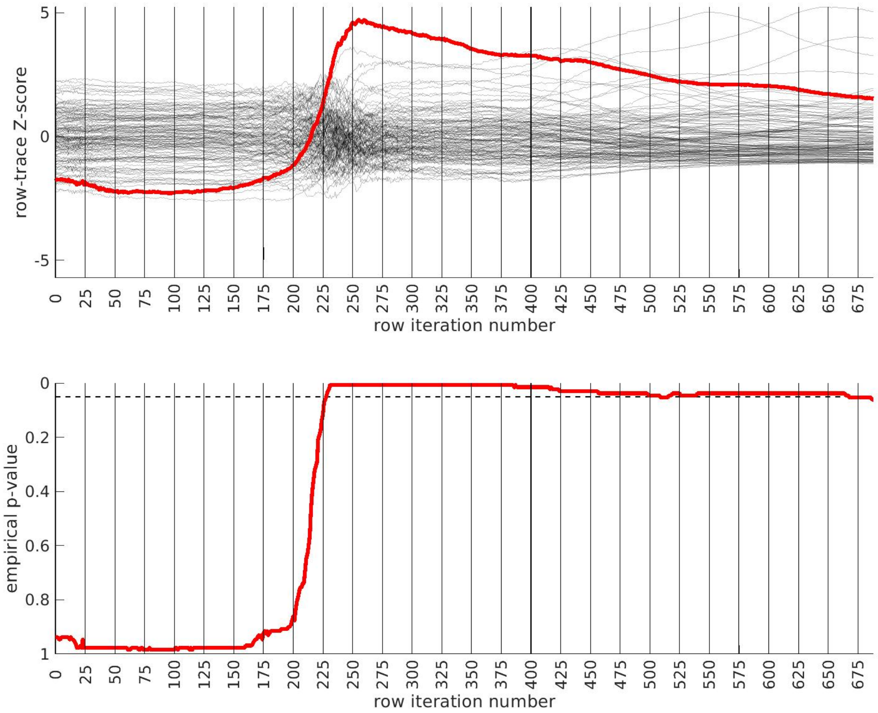

Fig 29. This figure is analogous to Fig 27, except that we consider arm-3 as a training-arm (rather than arm-1). Note that there is a distinguished peak to the red trace, indicating one bicluster. The overall replication p-value of this dominant bicluster is $p = 0.00010$ in arm-2, $p = 0.0025$ in arm-4, and $p = 0.0063$ in arm-1.

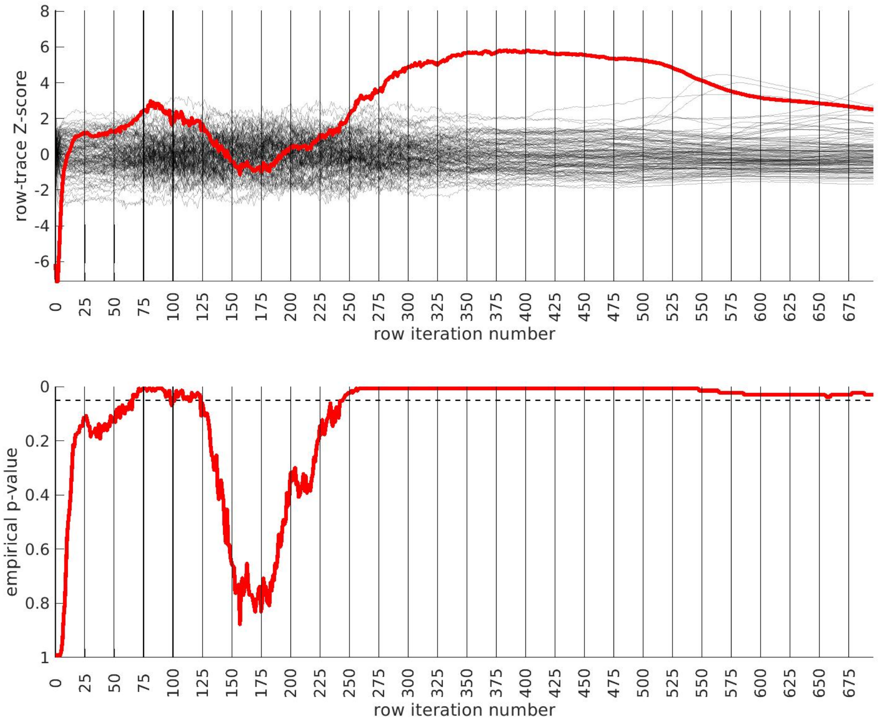

Fig 30. This figure is analogous to Fig 27, except that we consider arm-4 as a training-arm (rather than arm-1). Note that there are multiple distinguished peaks to the red trace, indicating (at least) one bicluster. The overall replication p-value of the dominant bicluster indicated by this trace is $p = 0.000025$ in arm-2, $p = 0.00013$ in arm-3, and $p = 0.040$ in arm-1.
